# I tweet, therefore I am: a systematic review on social media use and disorders of the social brain

**DOI:** 10.1186/s12888-025-06528-6

**Published:** 2025-02-03

**Authors:** Nancy Yang, Bernard Crespi

**Affiliations:** Department of Biological Sciences, Simon Fraser University, Burnaby, BC Canada

**Keywords:** Social media, Autism, Psychosis, Schizophrenia, Narcissism, Embodiment, Ipseity, Self-perception, Body dysmorphia, Delusion

## Abstract

With rapid technological advances, social media has become an everyday form of human social interactions. For the first time in evolutionary history, people can now interact in virtual spaces where temporal, spatial, and embodied cues are decoupled from one another. What implications do these recent changes have for socio-cognitive phenotypes and mental disorders? We have conducted a systematic review on the relationships between social media use and mental disorders involving the social brain. The main findings indicate evidence of increased social media usage in individuals with psychotic spectrum phenotypes and especially among individuals with disorders characterized by alterations in the basic self, most notably narcissism, body dysmorphism, and eating disorders. These findings can be understood in the context of a new conceptual model, referred to here as ‘Delusion Amplification by Social Media’, whereby this suite of disorders and symptoms centrally involves forms of mentalistic delusions, linked with altered perception and perpetuation of distorted manifestations of the self, that are enabled and exacerbated by social media. In particular, an underdeveloped and incoherent sense of self, in conjunction with ‘real life’ social isolation that inhibits identify formation and facilitates virtual social interactions, may lead to use of social media to generate and maintain a more or less delusional sense of self identity. The delusions involved may be mental (as in narcissism and erotomania), or somatic (as in body dysmorphic disorder and eating disorders, encompassing either the entire body or specific body parts). In each case, the virtual nature of social media facilitates the delusionality because the self is defined and bolstered in this highly mentalistic environment, where real-life exposure of the delusion can be largely avoided. Current evidence also suggests that increased social media usage, via its disembodied and isolative nature, may be associated with psychotic spectrum phenotypes, especially delusionality, by the decoupling of inter and intra-corporeal cues integral to shared reality testing, leading to the blurring of self-other boundaries.

**Supplementary Information:**

The online version contains supplementary material available at 10.1186/s12888-025-06528-6.

“The other’s gaze decentralizes my world” (Fuchs, 2002)

## Introduction

Since the advent of social media platforms, more and more human social interactions have been moving online. Over two billion people are active social media users worldwide, and more than 3.5 billion people have access to a mobile device [1]. In 2020, 94% of Canadian adults reported having at least one account on a social media platform [2]. Social media is also increasingly integrated into people’s social lives; for example, 97% of teenagers in the United States use the internet daily [3]. To date, approximately 80% and 69% of adults in the United States use YouTube and Facebook, respectively [4]. Seventy percent of Facebook users report accessing the site daily, and half of users report using it several times a day [4].

Social media sites have also become extremely popular applications within which people interact socially. Regular social media users report using Facebook as a “place to interact and socialize”, where they “have more contact with people via social media than face to face” and that “social media gives them a social life” [5]. Indeed, as the technology advances, more and more human interactions involve virtual means such as instant messaging, live video streaming, video calls, status updates, and virtual social networks with merging newsfeed and friend/follower requests. What are the psychological and psychiatric implications of the novel environments that social media usage creates? How does psychological makeup influence social media use, and how is social media use associated with risks and forms of psychological disorders? Despite the popularity of social media, the relationships of psychological phenotypes with patterns of social media usage remain understudied and little understood.

Virtualization of social interactions is expected to affect human social behaviors in a suite of domains, including but not limited to the construction of identities, the sharing of mental spaces, joint attention, eye contact, social interaction dynamics, relationships, and monitoring of social status (Table 1). Almost all virtual social processes can be asynchronous, entirely dissociated from physical embodiment, and free from temporal or geographic constraints. For example, whereas a traditional face-to-face conversation would involve two people in the same room talking, interpreting, and synchronizing to each other’s body language, pace of speech, and eye contact, a virtual “conversation” can now be enacted with asynchronous exchanges of text messages with anyone in the world, replete with memes and emojis to establish a virtual shared mental space and facial expressions in lieu of diverse physical cues. This disembodiment profoundly alters the nature of the social information that can be, and is, transmitted or exchanged.

**Table 1:** Comparisons between real-life and virtual social interactions

DOMAINS	REAL LIFE	VIRTUAL
Identity	- Continuous via iterated physical exchanges with other people - Shared (ex: doctor-patient, teacher-student, actor-audience) - Shared with other people by doing activities together - Identity co-created by shared narratives (ex: working on the same projects, sharing culture values or traditions)	- Discontinuous; can be entirely self-constructed by uploading videos, images, or other content online (ex: catfishing). Can be temporally disconnected with changes in online accounts - Can be “shared” virtually with likes, tags, comments or entirely self-curated (ex: impression management) - Narratives can be entirely self-generated via the use of videos, photos, or text (ex: creating a fake persona online by using photoshopped pictures or deepfake videos
Shared mental space	- Synchronized in tandem with physical actions (ex: body language, eye contact, vocalization) - Usually instantiated in physical embodiment (e.g.: nodding in agreement, dancing together, participating in shared religious rituals) in the same temporal and geographical spaces	- Completely disembodied, temporally and geographically asynchronous (e.g.: retweets, hashtags, Reddit upvotes, Twitter mobs) - Can be entirely unidirectional and/or done with an imaginary audience (e.g.: starting a hashtag on Twitter to make it trend, Zoom Yoga sessions)
Joint Attention	- Usually established by physical actions such as shared eye contact, pointing	- Simulated or “imagined” into existence (e.g.: posting a Tweet to an imagined virtual audience that may or may not exist)
Eye contact	- Shared by physically aligning pupil direction	- Can be entirely illusory (e.g.: curating LinkedIn image based on what an imagined employer wants to see) - Performative to an imagined audience, usually unidirectional (e.g.: making a TikTok video for a virtual audience)
Social Interaction Dynamics	- Immediate social feedback based on the other person’s body language, verbal response, or some physical cue	- Can be asynchronous, and thus largely rely on the users’ imagined or anticipation of what the other social media user is thinking/feeling (ex: Read Receipt Anxiety Syndrome)
Relationships	- Largely reciprocal, validated based on iterated physical social interactions	- Can be entirely unidirectional and without continuity (e.g.: following Twitter account anonymously) - Can be entirely illusory (e.g.: Facebook “friending” strangers)
Social Status Monitoring	- “Keeping up with the Joneses” - Done by comparing self with family, neighbours, colleagues, people in real life	- “Fear of Missing Out” - Done by comparing self with people on newsfeed online

In virtual domains, most social processes also become discontinuous. Thus, unlike in a typical physical social interaction, where a person constructs and maintains their identity based on iterated interactions in shared temporal and physical situations (e.g., work, school, family, and friend networks), digital identities can be self-generated through selective curation of videos, pictures, narratives and timelines, adapted to any online situation, and easily discarded and reinvented with the adoption of new, or multiple, digital accounts (e.g. [6]). The increasing popularity of following peoples’ social accounts also generates an evolutionarily novel situation where anyone, anywhere can make “social contact” and share in each other’s social lives by “participating” in one another’s social media feeds without ever having met face to face. The rise in social media platforms has thus not only changed how people socially interact (e.g., “lurking” in someone’s timelines instead of talking to them to make contact), but it has also fundamentally altered the human social landscape by dissociating, disembodying, and, as discussed below, amplifying social-cognitive processes in evolutionarily unprecedented ways.

Perhaps most importantly, success in social-media domains (i.e., gaining views, ‘likes’, ‘friends’, and followers) appears to usually engender high abilities in mentalistic cognition – the capacities involved in inferring, simulating, and anticipating the mental and social wants and reactions of others. Broadly speaking, mentalistic cognition refers to a large set of socio-cognitive abilities including eye-contact, joint attention, theory-of-mind, social imagination, narrative-production, and verbal intelligence – all of which are integral for initiating and facilitating successful social interactions [7, 8]. Unlike in real life face-to-face interactions where one would be able to receive immediate social feedback via physical cues such as body language and facial expressions, the anonymized and atomized nature of online social media interactions means that one is constantly updating and adjusting one’s thoughts and activities based on an imagined virtual audience, which, for success, requires high levels of social imagination and accurate perspective taking.

For example, to make a successful TikTok video, a person needs to know, a priori, what topics are engaging, and how to convey them to an invisible audience whose feedback will not be available until after the video is posted. Since the audience is not physically present, content creators must be able to anticipate the location and direction of the virtual eyes (i.e., illusory eye contact), focus on the point of virtual joint attention (i.e., illusory joint attention, illusory intentionality), and position themselves and perform in front of the camera in such a way that the imaginary audience feels personally engaged with the individual. Thus, to be socially successful online, one needs to be exceptionally adept in imagining, adapting to, and anticipating the attentional and affective preferences of an imagined virtual audience – all abilities expected to cluster with high mentalistic skills. Such ‘successful’ social media use needs to be distinguished, however, from social media usage in general, which varies greatly in its prevalence (as quantified with questionnaires, as typically done) and in how it attracts and affects its users.

Given that most human mentalistic functions have evolved in face-to-face interactions, what are the expected social-psychological implications of such wide adoption of virtual social technology? In this article we describe and evaluate the hypothesis that social media exaggerates and enhances mentalistic functions, that are over-developed, in the extreme, in so-called psychotic spectrum disorders (i.e., schizophrenia, schizotypal personality disorder, bipolar disorder I, affective psychosis, borderline personality disorder, and related conditions). This hypothesis is based in part on Crespi and Badcock’s [7] model of social brain variation and disorders, which posits that social cognition exists along a continuum, with normality at the centre, and grading towards increased mentalistic cognition in one direction, with typicality in the middle, and with decreased mechanistic cognition, involving higher levels of non-social, ‘mechanistic’ cognition regarding inanimate objects and systems [7], in the other direction.

How might autism-related traits, in contrast to mentalistic traits, be expected to interact with social-media environments? Autism-spectrum traits are characterized by reduced expression of mentalistic cognition, such as decreased eye contact, decreased joint attention, and decreased social interests, plus enhanced mechanistic traits such as enhanced visuo-spatial skills, interest in objects and mechanical systems (i.e., train tables or spinning wheels on a truck), fixation on special interest topics, stereotyped behaviors, proclivity toward solitary activities or occupations, and literal language interpretation [8]. All of these traits are associated in some manner with processing regularities in physical systems, which operate via algorithmic rules where input and output can be systematically predicted via pattern recognition [9, 10]. Given this set of findings, higher levels of autistic traits should be associated with lower rates of social media usage, but higher rates of internet usage related to systems, technology and special interests. When individuals with autism or high in autism-related traits use social media, the pattern of social media usage should thus be more mechanistic and less mentalistic – that is, individuals with higher levels of autism-related traits should be more likely to use social media platforms to share topics of their special interests, which are more factually-based than socially-driven, and use social media as a way to avoid or substitute for, rather than extend, real-life social contact.

In contrast to autistic symptoms and traits, positive symptoms of psychotic conditions, such as schizophrenia and schizotypy, are characterized by hyper-developments in social cognitive phenotypes, such as feelings of surveillance (i.e., excessive or illusory eye contact), paranoia (i.e., excessive or illusory joint attention), conspiratorial thinking (i.e., excessive or illusory theory-of-mind or social narratives), delusions involving social threats (i.e., delusions of persecution), and unusual perceptions (including social hallucinations and delusions) [8, 11]. As elaborated above, mentalistic abilities appear to be required for social media success, as high levels of theory-of-mind are necessary for cultivating an online persona that is engaging and efficacious. ‘Healthy’ (non-clinical, personality-level) positive schizotypal traits such as high creativity and imagination, particularly traits that enhance as well as extend mentalistic trait expression, should thus be associated with greater social media usage. Higher social media usage may also, however, be associated with psychotic spectrum traits that are facilitated and enabled by virtual social environments, as illustrated in Tables 1 and 2.

**Table 2:** Relationships between social cognitive traits, social media usage, and potential psychotic spectrum pathologies

Social Cognitive Trait	Virtual Equivalent	Potential pathology in individuals predisposed to psychotic traits
- social monitoring: eye contact, joint attention	- “Viewer” or “currently watching” counts on social media sites - “Read receipts” on instant messaging apps - Notifications that someone has viewed a profile	- Paranoia/conspiratorial thinking (illusory eye contact/joint attention) - Feelings of surveillance
- Interpersonal relationships (friends/acquaintance/extend-ed group members)	- Facebook “friends”, Twitter followers, Instagram followers	- Illusory fantasy relationships (e.g. erotomania, persecution, conspiratorial thinking)
- self-referential thinking	- Retweets, “likes” on Facebook posts - “Smart” algorithms on social media sites designed to show curated content consistent with users’ previous browsing history. Predictive analytics are also used to generate content that would be consistent with the users’ interests	- Magical thinking, ideas of reference (delusions of reference), erotomania, delusions of persecution and conspiracies, thought-broadcasting
- social imagination, narrative production	- Internet forums with shared interests - Virtual networks that are self-reinforcing based on shared ideas, beliefs, or politics (i.e. echo chambers)	- Folie à deux (or folie à million) - Social delusions (conspiratorial delusion, persecutory delusions, erotomania)
- self perception/self-embodiment based on iterated social exchanges and feedback and sensory stimulation that include in-person tactile and other non-verbal cues	- Virtual avatars in place of physical bodies - Online personas in place of physical identities - Selfies, filters, curated self-based images on Instagram and other image-sharing sites - Co-creation of digital identifies via Instagram “stories”, likes, comments, and tags	- Disorders characterized by perturbations in self-perception or somatic delusions (e.g.: body dysmorphic disorder, anorexia) - Disorders of disembodiment (derealization, depersonalization) - Excessive extended self (e.g. narcissism)

Based on these considerations, the following a set of predictions can be derived:

1. The different domains and traits of mentalistic cognition will manifest in particular, relevant aspects of social media use, in non-clinical, and grading into clinical, populations;
2. Higher levels of social media usage should be associated with an increased prevalence of psychotic spectrum traits and disorders; and
3. Higher levels of autistic traits should be associated with lower social media usage, and internet usage that is geared towards less-mentalistic cognition.

In this paper, these hypotheses were evaluated by adopting both trait-based and disorder-based approaches in systematic literature reviews within the relevant domains. In the trait-based analysis, we first reviewed the connections between specific psychological phenomenon and social media use, in the context of mentalistic cognition. We do not provide in-depth discussion of anxiety or depression and social media, because they have been reviewed extensively elsewhere (e.g., [12]). In the disorder-based analysis, we analyzed the relationships between patterns of social media usage among individuals exhibiting autism spectrum and psychotic spectrum conditions, with particular emphasis on disorders involving positive schizotypal or psychotic spectrum traits. We focus in particular on why some psychiatric disorders involve especially pronounced use of social media, while others do not.

## Methods

### Search strategies

We performed systematic reviews via the framework outlined by the Preferred Reporting Items for Systematic reviews and Meta-Analysis (PRISMA) statement. The review was conducted independently by the author Nancy Yang who performed the systematic search, with no automation tools. Systematic searches were conducted by combining the main relevant search term “social media” OR “facebook” OR “twitter” OR “instagram” OR “snapchat” OR “tumblr” OR “social networking” with each of the following key terms with the “AND” operator: “schizophrenia”, “psychosis”, “psychoses”, “psychotic disorder”, “schizophrenic disorder”, “paranoia”, “paranoid”, “persecutory delusion”, “bipolar disorder”, “bipolar I”, “bipolar II”, “manic depression”, “bipolar affective disorder”, “bipolar depression”, “body dysmorphia”, “body dysmorphic disorder”, “autism”, “ASD”, “autism spectrum disorder”, “Asperger’s”, “Asperger’s syndrome” “autistic disorder”, “eating disorder”, “anorexia”, “borderline personality disorder”, “bpd”, “emotionally unstable personality disorder” or “histrionic personality disorder”. “hpd”, “histrionic traits”, “dissociation”, “depersonalization”, “narcissism”, “narcissistic”, “narcissist”, “narcissistic personality disorder”, “erotomania”. Additional results were manually gathered from searching reference lists of existing reviews. The initial search yielded a total of 2623 studies from both PsycInfo (*N* = 1587) and PubMed (*N* = 1036).

### Inclusion criteria

A quantitative (including data analysis) or qualitative (including descriptions or observations without data analysis) study was considered for inclusion if it described data on at least one type of social media usage (or associated internet usage) with a psychotic spectrum trait or disorder (broadly construed, as noted above), or autism spectrum disorder. Qualitative studies, such as case studies, are especially useful for providing illustrative examples and for contextualizing the results from other work. Papers were excluded if quantitative studies did not include a control group for analysis, or if they involved editorials, commentaries, or addressed social media usage in the context of a clinical application (i.e., virtual mental health apps) or educational tools. Only papers published in English were selected, and all relevant papers were subjected to Google Scholar searches to find more-recent papers that cited them, and that also fit the inclusion criterion. As Facebook was introduced to the mainstream in 2004, we included papers published from 2004 to 2022, with February 28th, 2023 being the last date of search.

### Data extraction strategy

**Fig. 1:**
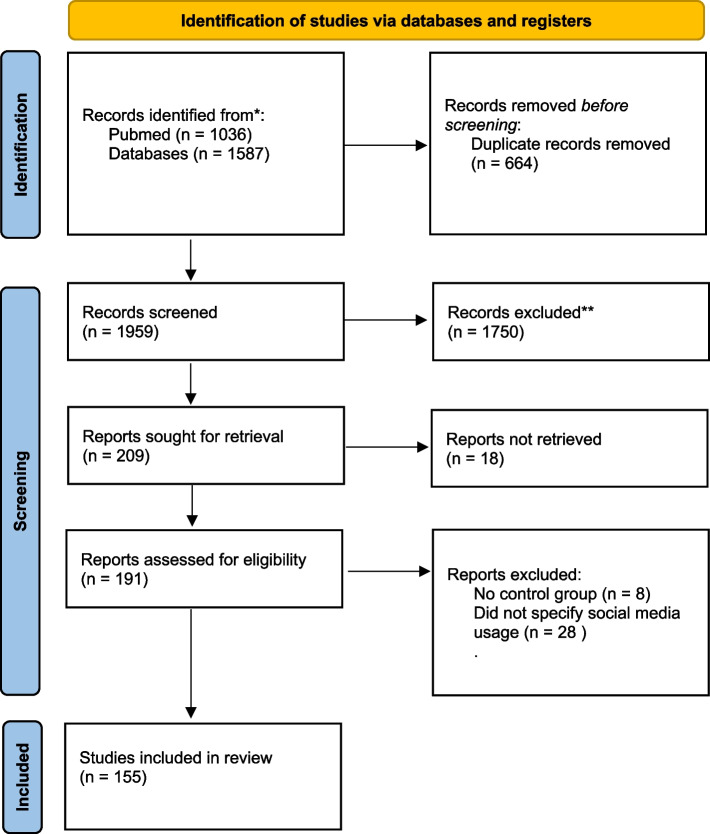
Flow-chart of the PRISMA-guided literature review on social media and psychotic-affective spectrum conditions and autism

Figure 1 describes the Preferred Reporting Items for Systematic Reviews and Meta-Analyses flow diagram of the study selection process. A total of 2623 papers was initially identified in the database search. 664 studies were removed as duplicates. The screening process involved scanning the titles and abstracts of each paper for relevance. This stage involved 1750 papers being excluded. Consequently, a total of 209 studies was sought for full-text retrieval. Eighteen papers were not retrievable and were discarded from further screening. Finally, 191 papers were assessed for inclusion based on their full text content. Papers were excluded for the following criteria: (1) if they were a quantitative study investigating general internet, smartphone, or online activity but did not specify whether social media activity was included (*n* = 28), or (2) had no control group in quantitative study (*n* = 8). In sum, a total of 155 articles was included in the review. No qualitative studies (e.g., case reports) that met the inclusion criteria were excluded.

## Results

### Trait-level analyses of social media usage

#### Patterns of social media usage in relation to different mentalistic domains

Social media as considered here is not a homogenous construct but a conglomeration of online functionalities that includes and emphasizes different social functions, depending on the nature of the site in question. Such media include, for example, Facebook, Whatsapp, Instagram, TikTok, WeChat, Instagram, Messenger, LinkedIn, Telegram, Snapchat, and Youtube. These platforms are diverse yet all include some elements of social cognition, social interaction, social engagement or social connection. For example, although Reddit and Instagram are both considered as “social media”, the former is more solitary and text-based, and involves asynchronous discussions dedicated to special interest subforums (subreddits) versus the image-driven Instagram, which is more geared toward curation of a self-narrative via videos, “stories”, and images with filters and other imaging-editing tools. Some sites also involve forms of entertainment, considered to elicit passive watching, although in many cases these involve watching other people and engaging socially with them in a detached way (e.g., by imagining their social lives). All such sites are ‘social’ in that they involve one person interacting with another person or their virtual incarnation, such that one or both are affected in some cognitive or affective way. Thus, social media usage patterns can be divided in terms of their correspondence to real-life socio-cognitive traits, and potential psychiatric disorders and traits that may be associated with usage, with particular attention to how different social media sites may affect different mentalistic functions.

We thus considered the following trait-based domains: (1) social monitoring and paranoia, (2) sexual relationships and erotomania, (3) reality perception, reality distortion and psychosis, and (4) dissociation and depersonalization. We also considered these domains, and the disorders associated with them, in the contexts of self-perception and self-embodiment, and self or body image. Each socio-cognitive trait and its corresponding virtual equivalent, as well as relevant pathologies, is discussed in detail below.

#### Social monitoring and paranoia

Seven papers fit the criteria for social media usage in relation to paranoia or social surveillance. As expected, the currently available data indicate that social media usage may enhance feelings of social monitoring. Four studies indicate that social media usage may contribute or exacerbate feelings of surveillance, even paranoia [13–16]. Two studies revealed that the asynchronous nature of communicating on social media may lead to hyper-mentalistic behaviours, such as worrying or imagining what the other party is thinking [17] (Schwartz M: The usage of Facebook as it relates to narcissism, self-esteem, and loneliness, unpublished thesis). Lastly, one study found that social media usage was positively associated with public self-consciousness [18], which has been linked to feelings of being watched [19].

Several lines of evidence suggest that social media may exacerbate feeling of social surveillance via its constant status updates and unpredictability of feedback. Thus, for example, young adults have reported curating their social media profiles and privacy settings according to the needs of an imagined audience (“you never really know who’s looking”) [13]. Problematic social media has also been associated with paranoia in a sample of adolescents [16]. Gill [14] interviewed young adults on their social media use, and found that women frequently reported feelings of being watched and judged on social media (e.g., feelings of being “stared at”), as well as reporting implicit expectations that their online content may be screen-saved and criticized. Furthermore, public self-consciousness (i.e., the outward displays of self as a social object) has been shown to be positively associated with a higher frequency of posting photos, replying to comments on photos, and resharing photos on social media, in a sample of South Korean university students [18]. Emotional venting, and viewing profiles of users who were not “friends” on social media, have also been associated with subsequent increases in paranoia [15].

‘Read receipts’, electronic notifications that a sent message has been opened and “read” by the recipient, have been widely adopted across social media platforms. A qualitative study found that university students often worry about what the other person is thinking when their messages are read but not responded to (“I think it makes people insecure… You have time to overthink about that situation… until the person responds”) [17]. One study presented participants with a sample text message conversation and asked them why a message was left on “read” with no response; most participants inferred that the non-response was intentional, and that a non-response constituted a rejection even when no explicit rejection was made (Schwartz M: The usage of Facebook as it relates to narcissism, self-esteem, and loneliness, unpublished thesis). These diverse lines of evidence suggest that the asynchronous and disembodied nature of social media has created a ubiquitous sense of remote surveillance, that may exacerbate feelings of social anxiety and paranoia, via excessive perceived virtual surveillance and social ambiguity.

#### Sexual relationships and erotomania

Five papers, discussing seven case studies in total, met the criteria for social media usage in relation to erotomaniac delusions (Supplementary Table 1). Two of the five papers observed that the patients with erotomaniac delusions had personal or family-related prior psychiatric histories involved psychotic spectrum disorders [20, 21]. Finally, four of the five papers reported that patients with erotomaniac delusions tended to also experience ideas of reference and magical thinking, mis-interpreting or over-interpreting hidden meanings in their love object’s online posting even when none existed. Taken together, the current data support the idea that social media usage may help to generate or sustain erotomaniac delusions in individuals with psychotic traits, particularly those involving hyper-mentalizing.

How might social media facilitate the development of erotomaniac delusions? Before the advent of online social media, human courtship and sexual relationships were initiated and built via some combination of face-to-face physical interactions with arrangements made by family or friends. The virtualization of social media is expected to alter the nature of such relationships in several important ways, potentially leading to enhanced risks for several forms of pathology.

First, as more relationships are virtualized, the incidence of erotomaniac delusions is expected to increase, as initiations of romantic or sexual encounters can become largely or entirely one-sided and based on illusory interest. Once referred as “psychose passionelle” [22], erotomania is a delusion wherein the individual, usually a young woman, believes that another person, usually of higher social status or unattainable in some other way, is in love with them despite an absence of evidence. Erotomania has thus been considered as an “excessive theory-of-mind” delusion where the individual becomes infatuated with impossible love objects [23], with mis- or over-attributing mental states of reciprocated love to others. Erotomania also commonly co-occurs with other psychotic disorders, including persecutory delusional disorder, schizophrenia [24], paranoid psychosis [25], bipolar disorder, and schizoaffective disorder [26].

Second, social media has removed or reduced physical and temporal barriers on mate seeking, which means that previously unattainable individuals (e.g., Hollywood stars) may appear accessible as prospective mates, since anyone can initiate contact via virtual means. The rise of ‘influencer culture’ on social media may also encourage more intimate experiences of ‘parasocial’ relationships; that is, illusory and one-way relationships where the viewer or social media user perceives the social media personality as a “friend” despite a lack of real-life interactions. In line with this view, exposure to, and interacting with, social media personas increase the strength of perceived parasocial attachment [27–30] and attraction [31]. These data suggest that even mere exposure to virtual personas can increase feelings of emotional attachment in neurotypical and healthy individuals, with increased exposure potentially leading to elevated risk of triggering forms of erotomania in vulnerable individuals.

Third, the typical patient with erotomania has been described as timid, sensitive, and socially isolated, experiencing difficulties with interpersonal attachments, and with tendencies towards ideas of reference [32]. Erotomania is also a female-biased delusional disorder, with a female to male ratio of about 3:1 [33]. Accordingly, five out of seven published case studies of social media-related erotomania show that the patient was female [20, 34, 35]. The patient is typically socially isolated, using social media as the primary or sole source of social interactions [20, 21]. Consistent across studies, patients also experience delusions of reference and perceived special hidden meanings in their love objects’ social media updates as “evidence” of their illusory love relationships [20, 21, 36]. Under a model of excessive mentalistic virtualization of socio-sexual interactions, high levels of social media usage may facilitate erotomaniac delusions in individuals who are socially isolated, exhibit impaired reality testing, lack real-life intimate relationships, and are prone to positive schizotypal traits such as excessive ideas of reference and magical thinking – all characteristics of the typical erotomania patient as described in the case studies in Supplementary Table 1.

#### Reality perception, reality distortion and psychosis

Thirteen papers met the screening criteria for social media usage and psychosis. Seven papers report case studies of patients suffering from social media or internet-related paranoia or delusions of persecution [37–43]. One paper reported a case study of an individual suffering from “hacking” delusion [44], which may be considered as the virtual equivalent of persecutorial delusion where individual privacy is lost. Three papers reported case studies of individuals who experience the delusion of having one’s sense of movements and agency being externalized to an online presence [45–47]. One article described a patient suffering from a delusion of online thought broadcasting [48]. Lastly, one paper documented the case study of a patient hearing commanding hallucinations through the internet [49]. Notably, all case studies discussed in the 13 papers have documented psychotic themes where there is a loss of the boundary between the self and non-self, which may be enhanced by the disembodied nature of internet or social media usage.

Reality distortion in psychosis includes magical thinking, ideas of reference (delusions of reference), thought broadcasting, and conspiratorial and persecutorial delusions, among other related phenomena. Increased immersion in virtual social media environments may be conducive to such psychotic cognition by altering reality perception in several ways. First, most social media platforms employ various forms of hidden website trackers that follow, monitor, and analyze users’ behaviors across a variety of websites to generate individualized ads and newsfeed to increase user engagement. The automatic curation of online content tailored to each users’ activity may promote the development of ideas of reference, as content is automatically and spontaneously updated “just-so” to each users’ previous activity [37]. Furthermore, the sense of disembodiment, blurring of self-other and private-public boundaries in online spaces, and lack of physical non-verbal cues may also increase feelings of mistrust, even paranoia, as online communications tend to be more ambiguous than face-to-face ones, and thus are more open to misinterpretation [43].

The increasing deployment of smart algorithms in social media platforms may also increase levels of hyper-mentalizing. For example, Twitter (now ‘X’) algorithms track users’ top-engaged accounts and rank them accordingly, curating the news feed in such a way that the users’ most-engaged account is shown first, which may heighten feelings of surveillance and delusions of thought-broadcasting as the website just “knows” what the user is most interested in. Indeed, paranoia is negatively associated with attitudes toward purchasing online, a finding that may be mediated by general distrust of advertisers’ use of subliminal messages [50]. Over time, the constant exposure to the individualized content may increase feelings of suspiciousness or paranoia that “someone” is watching them, and may serve to reinforce or exacerbate an individual’s underlying psychotic spectrum traits. Numerous case studies of social media or internet-related psychosis have been reported in the literature, that appear to derive in part from these considerations (Supplementary Table 2).

Several common themes link these cases of internet or social media-related psychotic traits. First, several case studies have observed that patients were typically socially isolated and spent time online in lieu of real-life relationships [37, 43, 45]. Second, feelings of surveillance (i.e., being monitored, tracked, followed) or externally controlled (e.g., hacked) were reported in the majority of case studies [38–42, 44, 47, 48]. For example, one case study (of Patient ‘A’) observed that the patient reported that people were following his activities online, and that a popular search engine was tracking his activities, as the name of the search engine shared the first two letters of his first time (“Al in” “Altavista.com”) [39]. Although the patient had cited a bizarre and implausible reason for being tracked online (i.e., name similarity) [39], modern search engines do monitor and curate results based on user activity, which may reinforce ideas of reference in high-risk individuals already predisposed to psychotic traits. Thus, the self-reinforcing nature of internet searches and smart algorithms may strengthen budding delusional beliefs. For example, patient W.L., who had a history of bipolar disorder, was already experiencing intrusive thoughts and feelings of suspiciousness when she started searching for the term “phenylalanine” online. When W.L. came across a webpage on an Aramaic system for divining special meaning from numbers, she interpreted it as secret information about the “Al-Qaeda network” and became paranoid that she was being monitored and tracked [38]. Likewise, patient K.D., who had previously consulted a physician for feelings of suspiciousness, developed the persecutory delusion that a secret organization was after him and his family, citing that the secret organization was behind hidden sections of several international companies’ websites [38]. Given that most websites do contain hidden sections that are not accessible to the general public, the uncertainty between private-public boundaries in online spaces may increase feelings of social surveillance to pathological levels in individuals who are already prone to paranoid ideation, as illustrated in the examples above.

Case reports have also noted that increased exposure to social media may lead to ideas of reference in individuals predisposed to psychosis [43]. In all three case studies reported by Nitzan et al. [43], the patient started experiencing psychotic symptoms, particularly delusions of reference, as they increased their social media usage to supplement the lack of interpersonal intimacy in their lives. In two of the three case reports, the patients started perceiving special hidden meanings in their social media newsfeeds and “friends” online messages with increasing social media use [43]. Given that most social media newsfeeds show users content that they are most “interested” in, the self-reinforcing cycle of smart algorithms may provide positive feedback that strengthens delusions of reference in users predisposed to psychotic-affective traits.

#### Dissociation and depersonalization

Four papers met the screening criteria for dissociation or depersonalization and social media usage. Two articles indicate that excessive social media or internet use are associated with increased levels of dissociative experiences [51, 52]. One study found that exposure to virtual reality environments can increase subsequent feelings of dissociation [53]. One study also noted that increased virtual social activities (i.e. video game, video meetings) have been positively associated with depersonalization experiences [54]. Given the novelty of virtual realities (i.e. video meetings) replacing in-person social activities, studies on the relationships of social media with dissociation are sparse. However, the available data indicates that increased virtual social interactions in lieu of real-life ones may be associated with increased levels of dissociation and depersonalization.

Dissociation, the experience of disconnections between sense of self, thoughts, memories, and emotions and detachment from the bodily self, personal agency, and objective reality, represents an imagination-based psychological experience that is both a typical experience in everyday life (e.g., in daydreaming, or being so totally immersed in reading a book as to lose track of one’s surroundings), and, in excessive form, a feature of positive symptoms of psychosis [55]. Use of social media is an intrinsically dissociative experience, given that the individual is generally mentally detached from their current physical and bodily surroundings as they focus on the online content at hand. Indeed, several studies have linked dissociation with social media use or other internet-based activities. For example, problematic social media usage and excessive Internet use have been associated with higher levels of dissociative experiences [51, 52]. Exposure to virtual reality environments has been found to increase subsequent feelings of dissociation, as well as diminish feelings of real-life presence in objective reality, in a sample of healthy college students [53]. Moreover, increased participation in other virtual activities such as video gaming and virtual meetings has also been positively associated with depersonalization experiences [54]. Given that social media usage induces dissociative symptoms in healthy individuals, excessive social media usage may be associated with more pronounced symptoms of dissociation, involving aspects of psychosis, among individuals predisposed to psychotic traits and socially isolated from the ‘real-life’ social world.

### Disorder-level analysis of social media usage and social brain disorders

We discuss a broad range of disorders, and it is important to bear in mind that, despite their DSM-based definitions, each of them is diverse in the symptoms displayed by each individual. Such variability emphasizes the need to avoid stereotyping, and to consider psychiatric disorders as due to many genetic, environmental and interactive causes.

#### Psychotic spectrum disorders and general social media usage

Psychosis involves reality distortions: the loss of abilities to differentiate what is real and what is not. Given that the psychotic spectrum encapsulates a wide range of conditions, we summarize each condition’s relationship to this spectrum, and to social media usage, in detail below.

#### Schizotypy and schizophrenia

Schizophrenia involves some combination of positive (psychotic), disorganized (cognitive), and negative (social withdrawal, anhedonia, and other traits losses or reductions) symptoms [56]. Schizotypal personality disorder, which involves similar but less pronounced symptoms, is characterized by ideas of reference, magical thinking, odd thinking or speech, unusual experiences, bodily illusions, suspiciousness or paranoid ideation, constricted or inappropriate affect, and social anxiety that does not diminish with familiarity [57]. Given that one aspect of positive schizotypal traits is social anxiety, individuals higher in schizotypal traits might be expected to exhibit greater social media use, in lieu of in-person social interactions, given its more impersonal nature.

Of the five studies addressing schizotypal personality and social media usage, three found positive relationships between schizotypal traits and online social activities [58–60] (Table 3). For example, two studies have found positive relationships of schizotypal traits with chatroom participation [58] or Facebook usage [59]. One study has detected a positive association between problematic internet usage and psychotic-like experiences [60]. Likewise, internet addiction symptoms have been linked to disorganized schizotypy [59].

**Table 3:** Relationships between social media usage and schizotypal traits

References	Methods	Main Findings
Mittal et al. 2007 [60]	Self-reports of daily Internet use in adolescents with SPD (*N* = 19), a control group with other personality disorder (*N* = 22) and a healthy control group (*N* = 28)	- Participants with SPD reported significantly less real-life social interactions and more online social interactions (ex: online gaming, online chat rooms) than controls - SPD symptom severity positively correlated with chat room participation, cooperative internet gaming and email use
Mittal et al. 2013 [58]	− 170 young adults (mean age = 19.1) were followed for two months. They were categorized into two groups: (1) steady/improved course of psychotic-like experiences (PLE) and (2) those showing increases in psychotic-like experiences. Psychotic-like experiences were measured with the Prodromal Questionnaire-Brief. - PLE improved/constant group (127 adults, 81 males, 46 females, mean age = 18.9). PLE increase group (43 adults, 26 males, 17 females, mean age = 19.7) - Internet addiction and a factor “reality substitute” (i.e. the extent to which the individual perceives the internet as another reality and over depend on it for relieving real life problems) were examined within and between the two groups	- Although both groups reported similar levels of internet addiction and reality substitute at baseline, the PLE improved/constant group showed longitudinal declines in both domains whereas PLE-Increase group’s reported level remained constant - Psychotic-like experiences moderately positively correlated with problematic internet usage; magnitude of association with Reality Substitute for the PLE-Increase group grew significantly over time
Massaro et al. 2022 [59]	− 270 undergraduate students (age range = 18–30, 50% female and 50% male) completed the following questionnaires: demographics and health questionnaire, Facebook Use Scales, Internet Addiction Test, Schizotypal Personality Questionnaire Brief-Revised, Generalized Anxiety Disorder 7-item Scale, Personal Health Questionnaire Depression Scale (PHQ-8)	- Schizotypy total scores predicted internet addiction behavior and frequency of Facebook use - Disorganized schizotypy was the strongest predicator of internet addiction symptoms
Hogg 2009 (Hogg JLC: Impact of personality on communication: an MMPI-2 study of African American college students and their choice in the digital communications age, unpublished)	− 159 African-American college students (126 females, 33 males, mean age = 21.6) completed the Minnesota Multiphasic Personality Inventory, the Saba-Penn Demographic Survey, the Cross Cultural Research Team Communications Questionnaire	- Participants who spent “excessive” time instant messaging were more likely to endorse hypersensitivity, suspiciousness, odd behaviors, unusual perceptions, and feeling of disconnection from reality
Tamás et al. 2022 [61]	− 717 individuals (186 males, mean age: 28.49, 531 females, mean age: 28.4) completed a demographic questionnaire, the Immersive Tendencies Questionnaire (ITQ), the Tellegen Abosprtion Scale, the Schizotypal Personality Questionnaire-Brief Revisited, Self-concept Clarity Scale, Mental Health Continuum Short Form, Bergen Facebook Addiction Scale, Problematic Internet Usage Questionnaire, and questions to assess social media usage frequency	- Higher scores in Involvement scale of the ITQ are associated with greater problematic Facebook usage, problematic internet behaviours, and higher schizotypal scores

Two other studies found more or less indirect evidence between schizotypy and social media usage. For example, one study has found that increased text messaging is associated with increased suspiciousness (Hogg JLC: Impact of personality on communication: an MMPI-2 study of African American college students and their choice in the digital communications age, unpublished). Another study has found that higher immersive tendencies may be associated with greater problematic Facebook usage, problematic internet behaviours, and high schizotypal scores [61]; however, direct relationships between problematic Facebook usage and schizotypal traits were not investigated.

Of the papers that investigated the relationships between social media usage and schizotypal traits or psychosis, one found increased social media usage linked to prediction of positive psychotic-like experiences in a sample of non-clinical individuals [62], whereas the other found that adults diagnosed with psychosis used social media less frequently compared to a control group [15].

Overall, as summarized in Table 3, current data suggest that schizotypal traits may be associated with social media usage. For example, Mittal et al. [58] found that adolescents diagnosed with Schizotypal Personality Disorder spent more time in online chat rooms compared to controls, and severity of schizotypal personality disorder symptoms were also positively correlated with cooperative online gaming, internet chat room, and email usage. Similarly, in a sample of young adult with psychotic-like experiences, problematic internet usage and reality substitution (the extent to which the individual perceives the online environment as another “reality” and immerses themselves in it) showed longitudinal decline in the group whose psychotic-like experiences improved or remained steady; in contrast, levels of problematic internet usage and reality substitution were constant in the group whose psychotic-like experiences increased [60]. Likewise, Massaro et al. [59] reported that schizotypy total scores predicted internet addiction behavior and frequency of Facebook use in a sample of undergraduate university students. In addition, Hogg (Hogg JLC: Impact of personality on communication: an MMPI-2 study of African American college students and their choice in the digital communications age, unpublished) found that college students who spent ‘excessive’ time instant messaging endorsed higher levels of suspiciousness, fearfulness, and dissociation from reality – all of which are common features of positive schizotypy.

Notably, Hogg (Hogg JLC: Impact of personality on communication: an MMPI-2 study of African American college students and their choice in the digital communications age, unpublished) found, among healthy college students, that spending 11-15 h a week on instant messaging and social media was associated with the highest level of psychological well-being. Risk to well-being increased when instant messaging dropped below or increased beyond this level. Furthermore, psychopathological symptoms (e.g., suspiciousness, rigidness in thought, peculiar perceptions, and dissociation from reality) were most strongly associated with instant messaging when it exceeded 26 h a week (Hogg JLC: Impact of personality on communication: an MMPI-2 study of African American college students and their choice in the digital communications age, unpublished). Finally, Tamás et al. [61] reported that higher scores on the Involvement scale of the Immersive Tendencies Questionnaire were associated with greater problematic Facebook usage, problematic internet behaviours, and higher schizotypy scores. The Involvement scale measures the degree to which individuals experience a sense of passive immersion while reading a book or playing computer games [61]. Given the above findings, positive schizotypal traits may be associated with greater social media usage due in part to the former’s dissociative nature.

These findings suggest that positive schizotypy may be associated with greater social media usage, with the effect moderated by different levels of immersion in social media. However, given that only one out of three available studies directly tested the relationships between positive schizotypy and social media (i.e., Facebook) usage [59], further research is needed.

Two studies investigated the relationship of social media usage among individuals with psychotic-like experiences or schizophrenia (Table 4 ). One study found that number of hours of social media use per day contributed significantly to the prediction of positive psychotic-like experiences, in a sample of non-clinical undergraduate students [62]. By contrast, adults diagnosed with psychosis used social media less, compared to the control group [15]. The same study also found that the following social media activities predicted increases in paranoia: 1) posting about feeling and emotional venting, 2) viewing profiles of people who were not “friends” on social media, (3) commenting on other peoples’ status updates. Although empirical studies on the relationships between social media usage and paranoia in patients with psychosis are thus lacking, current data suggest that social media activity, particularly those that imbue a virtual feeling of surveillance, may underlie decreased social media activity in individuals with clinical diagnosis of psychosis. By contrast, individuals with positive schizotypal traits, particularly those high in social anxiety and disorganized faculties, may gravitate toward greater social media or usage of virtual social media, possibly due to its relatively anonymous, distancing, and immersive nature of social engagements (Table 3).

**Table 4:** The relationship of social media usage with schizophrenia spectrum disorders

References	Methods	Main Findings
Fekih-Romdhane et al. 2021 [62]	The Positive Subscale of Community Assessment of Psychotic Experiences and the Arabic Social Media Addiction Scale (ASMAS) were administered to a total of 1007 college students (64.6% female; mean age = 21.9).	- Number of hours of social media use per day contributed significantly to the prediction of positive psychotic-like experiences (bizarre experiences, perceptual abnormalities, persecutory ideation, and magical thinking)
Berry et al. 2018 [15]	25 non-clinical controls (11 male, 14 female, mean age = 35.4) and 19 clinical individuals (7 male, 12 female, mean age = 33.7) with a diagnoses of schizophrenia spectrum disorder with psychosis completed self-assessment of social media use, perceived social rank, mood, self-esteem, and paranoia	- Participants diagnosed with psychosis used social media less compared to the non-clinical group, and were less likely to use Facebook than controls - The following social media activity predicted increases in paranoia: (1) posting about feeling and emotional venting, (2) viewing profiles of people who were not “friends” on social media, (3) commenting on other peoples’ status updates

#### Bipolar disorder

Bipolar disorders involve mania, hypomania, depression and mixed states, with varying degrees of severity, and forms of psychosis in Bipolar I [63]. Three studies met the search criteria for social media usage in individuals with bipolar spectrum disorders. Out of the three studies, one had found a positive relationship between clinical symptoms of bipolar-mania and Facebook usage [64]. Rydahl et al. [65] found that individuals with bipolar disorder were more likely to report social media “regret” behaviours compared to controls (e.g., “Writing private messages”, “Sending photos privately” and “Sending videos privately”), but direct relationships between bipolar disorder and social media usage were not investigated. Lastly, Martini et al. [66] found that patients with bipolar I or bipolar II disorder exhibited less familiarity with social networking sites and had fewer Facebook contacts than controls.

Given that mania episodes in bipolar I disorder are characterized by some hyper-mentalistic (e.g., paranoia, feeling of surveillance, delusions of reference) and hyper-social behaviors (e.g., sexual promiscuity, gregariousness), social media usage is expected to increase during manic phases of bipolar I disorder. As elaborated below, available studies suggest that increased manic symptoms in bipolar disorder may be associated with greater active social media usage [64] or other use of online media, in a way to mirror real-life manic behaviours [65]. However, Martini et al. [66] found that patients with bipolar I and bipolar II had fewer Facebook friends than controls. Given the few studies available on social media usage in bipolar spectrum disorder, no definite conclusions can be drawn.

#### Narcissistic personality disorder

Narcissistic personality disorder (NPD) is characterized by grandiose self-perception, need for admiration, interpersonally exploitative behaviors, and lack of empathy [67]. Narcissistic personality disorder is typically characterized by fantasies of grandeur, or the idea that the self is exaggerated to be unique and superior, although it can also involve forms of psychological vulnerability expressed in social hypersensitivity and defensiveness [68]. NPD is notably comorbid with several other psychotic/affective spectrum conditions, including paranoid personality disorder [69, 70], major depression [71, 72], and bipolar disorder [72–74].

A total of 70 papers met the screening criteria for social media usage in narcissistic personality disorder. The majority of these papers (sixty) show that narcissistic traits are positively associated with greater social media usage [75–93], greater social media usage for romantic purposes [94], greater production of content on social media [95], greater frequency of selfie-posting [96–105], selfie-editing [106], selfie-liking [107], Facebook friends [108–110] (Kojouri C: Using Facebook to self-enhance: narcissism and psychological outcomes, unpublished) and status updates [111–113], greater self-promotional content on social media [114, 115], problematic or addictive social media usage [116–120], greater self-promoting behaviors on Facebook [121], more interacting with Facebook photos [122], Facebook addiction [87, 123], internet addiction [124], greater Facebook intensity and personal involvement (i.e. “I want to express and present myself” [125], Facebook addiction symptoms [123, 126–128], risk factors for Facebook addiction [129], greater motivation of using social media for self-promotional purposes [130], and greater importance and involvement placed on feedback received online [131, 132].

In contrast to the findings above, seven studies reported that narcissistic traits were not associated with increased social media usage [133–136] (Schwartz M: The usage of Facebook as it relates to narcissism, self-esteem, and loneliness, unpublished thesis), increased numbers of Instagram followers [137], or number of selfies posted [138]. Pathological narcissism was also found not to be a significant predicator of selfie-engagement [139]. Three studies yielded mixed findings on narcissism and social media usage. For example, Eşkisu et al. [140] found that narcissism scores did not differ between those who with or without a Facebook account. However, the same study also found that narcissism scores were significantly higher for individuals who spent more than 3 h on Facebook and have more than 300 Facebook friends, compared to those who only use Facebook for less than one hour/day and had 151-300 friends. Lastly, Martin [141] found that higher vanity scores were associated with posting more athletic photos on Facebook in both sexes, but only men scoring low in vanity posted more attractive photos. The findings above suggest that, as social media platforms have become more mainstream, the relationships between social media usage and narcissism may emerge predominantly in the upper percentile of usage.

Central characteristics of NPD also include exhibitionism, entitlement, and excessive need for admiration [67], all of which are consistent with the mentalistic trait of social surveillance. Given that narcissism is characterized by excessive need for admiration and self-aggrandizing behaviors, higher narcissistic traits are expected to be associated with greater social media usage, as well as self-promotional behaviors, an expectation that is borne out by the reports in Supplementary Table 3.

In accordance with the attention-seeking behaviours of real-life narcissism, narcissism-exhibitionism is positively associated with frequency of Twitter usage, Facebook friends, and greater expression of emotions on social media [88]. Initial levels of problematic internet use predict subsequent levels of narcissism four months later, but only for those who used primarily visual-based forms of social media such as Instagram [119]. Individuals scoring higher on vanity also posted more athletic photos on Facebook [141]. Individuals with higher narcissistic traits were also more involved in feedback (e.g., comments and likes) received on their selfies, more observant of selfies that other people have posted, but not increased likelihood of providing feedback on other peoples’ selfies [132]. Individuals with higher narcissistic traits also regard self-posting as more positive and report higher motivation for posting selfies in the future [132]. Furthermore, social media usage is positively associated with narcissism and alexithymia, and negatively associated with empathy [89]. Taken together, these lines of evidence are consistent with the narcissistic real-life behaviours of being especially attuned to illusory social gaze, particularly involving admiration.

The findings noted above are consistent with reports that individuals high in narcissism tend to be more motivated to seek out situations of positive self-attention, such as gazing at oneself in the mirror, and increased preference of watching oneself on videotapes rather than watching videotapes of others [142]. Indeed, the top motive for taking selfies in a sample of undergraduate students were for narcissistic reasons (e.g., “I think I am attractive and have no problem sharing that”) [104], and for attention-seeking [105]. Grandiose narcissism has also been associated with frequencies of selfie-taking and selfie-posting, as well as experiencing more positive affect when taking selfies [99]. Considered together, the above lines of evidence indicate that the narcissistic pattern of social media usage is consistent with the real-life behaviours of the narcissistic personality - that is, that of the overinflated self-perception.

Although many articles have observed a consistent pattern of higher social media usage in narcissistic individuals, some findings observed no statistically significant relationships between the two [133–136]. Although some studies have found no significant relationships between narcissism and greater social media usage, the general pattern of use appears to be largely consistent with the selfie-oriented behaviors of real-life narcissism. For example, although Barry et al. [138] found no significant correlation between narcissism and total number of selfies, the same study also observed that vulnerable narcissism was positively associated with a higher proportion of posts that were ‘appearance selfies’ (pictures just of oneself). Conversely, grandiose narcissism was negatively associated with proportion of posts that were ‘affiliation selfies’ (i.e., selfies with other people) [138] - both of which are consistent with prior findings that hypersensitive (i.e. vulnerable) narcissism is positively associated with greater levels of self-objectification [143] and general narcissism with greater need to be at center of attention [144]. Interestingly, although Scott et al., [145] found no significant associations between narcissism and selfies, narcissism predicted likelihood of posting photos in the “pet” category. This finding is also consistent with prior observation that grandiose narcissism is associated with stronger attachment to “traditional” pets like dogs and cats [146]. Other studies have found no relationships between narcissism and number of Instagram followers [137], narcissism to frequency of Facebook checking or Facebook updates [140], or differences in narcissism between individuals who has a Facebook account versus those who don’t [140]. However, in the same study, Eskisu et al. [140] observed that narcissism scores were significantly higher for participants who spent more than three hours a day on Facebook vs. participants who spent less than an hour and had more than 300 Facebook friends versus those who had 151-300 friends [140]. Taken together, the current data suggest that the narcissistic pattern of social media usage largely mirror real-life narcissistic behaviours.

Finally, as summarized in Supplementary Table 3, selfie-taking and status updates are two of the main primary social media activities that have been consistently linked to higher narcissism across studies, demonstrating the self-oriented and performative nature of mainstream social media platforms.

Individuals high in narcissistic traits may also be attracted to social media platforms, as they allow for a large network of loose and impersonal relationships as well as various tools for self-enhancement [114]. For example, scores on the Grandiose-Exhibitionism dimension of narcissism predict self-promoting Facebook behaviors, Facebook friends count, and frequency of accepting strangers as Facebook friends [121], as well as placing greater importance on receiving responses online and looking popular on social media [131]. Grandiose narcissism has also been positively related to time spent on social media, frequency of status updates, number of friends/followers, and frequency of selfies posted on social media [81]. Facebook users were more likely to be narcissistic and extraverted than non-Facebook users [93]. As observed previously by Carpenter [121], patterns of social media usage in narcissism appear to represent “extensive self-presentation to as large as an audience as possible”, which is consistent with the current data showing that individuals with narcissism tend to use social media for self-promotional purposes (Supplementary Table 3). However, Frederick and Zhang [136] observed no significant relationships between narcissism and social media usage. Boursier et al., [139] has also found that pathological narcissism was not a significant predicator for selfie-engagement; rather, selfie-engagement predicted by body surveillance and positive selfie-expectancies about selfie-taking.

#### Borderline personality disorder

Borderline personality disorder is characterized by pervasive pattern of instability of interpersonal relationships, mood swings, labile affect, impulsivity, self-harm or suicidal behaviors, chronic feelings of emptiness, dissociative symptoms, and transient stress-related paranoia [147]. Borderline personality disorder frequently involves positive psychotic symptoms [148, 149]. Accordingly, by the hypotheses evaluated here, borderline personality disorder should be associated with increased social media usage.

Only two papers met the criteria for social media usage in borderline personality disorder.

Higher borderline personality disorder traits have thus been associated with greater frequency of posting on social media, greater self-reports of regret after posting on social media, greater likelihood of editing or deleting posts after posting, more frequency friending and unfriending behaviors on social media [150]. Borderline personality traits are also associated with increased cyberbullying behaviors in adolescents, particularly malicious social gossip [151], the production of which requires substantial mentalizing skills (e.g., social network monitoring, reputation tracking) [152].

#### Histrionic personality disorder

Histrionic personality disorder is characterized by excessive attention-seeking behavior, suggestibility, theatricality, being uncomfortable in situations when they are not the center of attention, and considering relationships to be more intimate than they really are [153]. In a sample of 26 patients with recent-onset bipolar spectrum disorder, histrionic personality disorder was found to be the top co-occurring personality disorder [154].

Three papers meet the criteria for histrionic personality disorder and social media usage.

Considering that most social media encourage self-displays (i.e., status updates, selfies), excessive theatricality and attention-seeking behaviors in individuals with histrionic personality disorder should be associated with greater social media usage, as well as greater self-display behaviors such as selfie-posting. Accordingly, histrionic personality traits have been associated with greater social media usage, addictive use of social media, and increased selfie-sharing [155]. In a sample of adolescents aged 14-18 years old, social media addiction has also been positively associated with histrionic personality belief (e.g., “I should be at the center of attention.“) and narcissistic personality beliefs (e.g., “Other people should satisfy my needs”) in both sexes [156]. Similarly, histrionic traits predicted number of selfies posted online, but in men only [157]. The currently available data thus supports the prediction of higher mentalistic traits (i.e., self-display to a virtual audience) in that histrionic personality disorder is associated with greater social media usage. However, given that there are only three available studies on social media usage in histrionic personality disorder, additional studies are needed.

#### Body perceptual disorders: body dysmorphic disorder and anorexia

Body dysmorphic disorder (BDD) involves pathological preoccupation with perceived negative aspects of one’s bodily appearance, and anorexia is an eating disorder that involves extreme fear of gaining weight, coupled with highly altered perception of overall body weight and image [158, 159]. Thirty-two papers met the criteria for social media usage in individuals with body dysmorphia disorder or anorexia. Of these, 17 studies found that higher social media usage was associated with increased body or weight dissatisfaction [160–168], internalization of the thin-ideal [169, 170], eating disorder symptoms [171–174], dysfunctional eating patterns [175]. Accordingly, Smith et al. [176] found that maladaptive Facebook usage predicted increases in over-eating episodes four weeks later. Five studies reported that online physical appearance comparison was associated with greater disordered eating [177], greater body dissatisfaction [178], drive for thinness [179] or restrained eating via body shame [180].

Three studies found that image-based social media activities may be positively associated with eating disorder symptoms [181–183]. In addition to image-based activities, receiving extremely negative feedback to one’s online status updates has been associated with subsequent increases in disordered eating concerns [184]. Taken together, all four papers indicate that increased engagement with social media content can be linked with greater body dissatisfaction and disordered eating symptoms.

In contrast to these results, two studies found no association between social media usage to body dissatisfaction or body image issues [185, 186], and four studies reported mixed findings on the role of social media usage and eating disorder symptoms. For example, Kim and Chock [187] found that viewing and commenting on online peers’ profiles, but not time spent on social media, were associated with increased body image concerns [187]. Similarly, Cohen et al. [188] found that it was not time spent on social media but following “health and fitness” Instagram accounts that was associated with drive for thinness. Tiggemann and Slater [189] found that number of Facebook friends, but not Facebook usage, prospectively predicted greater drive for thinness in a sample of adolescent girls. Cohen et al., [190] found that it was greater selfie investment, not but general social media usage, that was associated with increased body dissatisfaction and bulimia symptoms. Considered together, the current data suggest that it may not be general social media usage per se that is associated with increased symptoms of body dysmorphia or eating disorder symptoms, but a more specific factor, active online physical appearance comparison, that may instead be driving the links between social media usage and body image concerns.

Relationships of social media usage with body image disorders have been well documented in the literature [191]. However, how social media usage may be associated with body dysmorphia and anorexia as psychotic spectrum conditions has yet to be systematically explored. Both BDD and anorexia are characterized by extreme distortions in body self-image. Historically considered as prodromal to, or a variant of, schizophrenia [192–195], BDD is characterized in particular by excessive occupation with a slight or imagined physical flaw; consequently, the individual may engage in compulsive grooming or reassurance-seeking behaviors as to correct or hide the perceived flaw.

In contrast to individuals with BDD, individuals with anorexia perceives themselves to be excessively overweight, despite evidence to the contrary, and may restrict eating and compulsively exercise excessively to mitigate the self-perceived weight gain. As in BDD (Walker M:Associations between Facebook use and disordered eating in College Women, unpublished), individuals with anorexia also engage in excessive or compulsive body-checking behaviors such as examining their body or body parts in the mirror, checking if their thighs touch, or pinching the stomach to see if it is ‘excessively’ fat [196, 197].

Both BDD and anorexia involve several psychotic spectrum traits. For example, BDD is often characterized by ideas or delusions of reference, such that individuals with this condition are convinced that other people are mocking or noticing their physical flaws [198, 199], experiencing such distress that they may become housebound to avoid being seen [199, 200]. Schizophrenia also shows significant positive genetic correlations with anorexia and other eating disorders [201], indicating a shared genetic based, and BDD exhibits both delusional (psychotic) and somatic presentations, both with high frequencies [202].

Given that both BDD and anorexia are characterized by perturbations to body perception, increased social media usage may promote the expression of BDD and anorexia, as most social media platforms are image-based and encourage the use of multiple photo-editing tools that can be used to create unrealistic expectations of the statistically “normal” body proportions, as individuals compare their bodies to curated images seen on social media. In addition, individuals with BDD or anorexia also engage in compulsive and excessive mirror-checking as part of their body perceptual disorders; as such, given that most social media platforms are image-centric and “reflect” back its users visually based content, BDD, body dysmorphic traits and anorexia should also be positively associated with greater social media usage as virtual extensions of mirror-checking behaviors.

As described in Table 5, three out of four studies provide evidence that body dysmorphic traits may be associated with greater social media usage [203–205]. For example, Alsaidan et al., [203] found that body dysmorphic disorder was associated with greater time spent on Instagram and Snapchat, both image-based platforms with a wide plethora of picture and video-editing tools. Accordingly, Griffiths et al., [204] has found that muscularity dissatisfaction and eating disorder symptoms were stronger for image-centric platforms (e.g., Instagram) than for non-image-based platforms (e.g., Wordpress). Finally, social media addiction symptoms have been positively associated with muscle dysmorphia-related symptoms, eating disorder-related symptoms, psychopathological distress, and problematic alcohol use [205]. While one study did not find statistically significant relationships between Instagram use and body dysmorphic concerns [206], this study did identify an indirect connection between Instagram use and dysmorphic concerns through the concepts of ‘ideas of reference about laughing and commenting’ and ‘appearance-related comparisons, both of which involve mentalistic faculties of imaginary social surveillance and the illusory social gaze.

**Table 5:** Relationships of social media usage with body dysmorphic disorder

References	Methods	Main Findings
Alsaidan et al., 2020 [203]	- Cross sectional study conducted during January and February 2020 − 1010 participants completed the Body Dysmorphic Disorder Questionnaire (BDDQ) and social media usage questionnaire online. − 4.2% of the participants met the BDD diagnosis threshold (“BDD status”) via scores on the BDDQ	- BDD associated with spending more time on Snapchat and Instagram
Senín-Calderón et al., 2020 [206]	− 796 participants (mean age = 22.5; 54% women) completed the Dysmorphic Concern Questionnaire, Referential Thinking Scale, Difficulties in Emotion Regulation Scale, Physical Appearance Comparison Scale-Revised, and a questionnaire on Instagram use	- Although direct effects between Instagram use and dysmorphic concerns was not statistically significant, Instagram use was related to dysmorphic concerns via appearance-related comparisons, ideas of reference about “laughing, commenting,” and difficulties in emotion regulation
Griffiths et al., 2018 [204]	− 2,733 sexual minority men (mean age = 33.9) completed questionnaires on social media and dating app usage, Male Body Attitudes Scale–Revised (MBAS-R), Eating Disorders Examination Questionnaire Short (EDE-QS), and attitudes about anabolic steroid usage - Minority sexual orientations are categorized as followed: 68.4% exclusively gay/homosexual, 21.4% mostly gay/homosexual, 8.4% bisexual, 1.1% mostly straight/heterosexual, 0.7% “other”	- Higher social media usage, particularly Facebook, Instagram, and Snapchat, were associated with greater body image concerns and eating disorder symptoms - Associations between social media use and muscularity dissatisfaction and eating disorder symptoms were stronger for image-centric (ex: Instagram) than for nonimage-centric social media platforms (ex: Wordpress)
Imperatori et al., 2022 [205]	-721 young adults (504 females, mean age = 24.13) completed questionnaires on demographics, the Bergen Social Media Addiction Scale, the Eating Attitudes Test-26 (i.e. eating disorder symptoms), the Muscle Dysmorphic Disorder Inventory, the Brief Symptom Inventory (measures psychopathological symptoms), the Cut-Annoyed-Guilty-Eye (CAGE) questionnaire (i.e. problematic alcohol use)	- Social media addiction- symptoms were positively associated with muscle dysmorphia-related symptoms (i.e. extreme preoccupation with one’s appearance, muscularity, and compulsive physical exercise), eating disorder-related symptoms, psychopathological distress, and problematic alcohol use.

As summarized in Supplementary Table 4, increased social media usage has also been associated with increased eating disorder symptoms [171–174], greater body dissatisfaction [162, 166], weight and appearance dissatisfaction [164], preoccupation with body and food intake [175], internalization of the thin ideal [169], and dieting behavior [170]. Furthermore, compared to Facebook non-users, Facebook users score higher on the Internalization of thin idea, a body surveillance based drive for thinness [165].

Increased exposure to particularly image-centric social media platforms may be associated with greater body dissatisfaction and thus with eating pathology. For example, Cohen et al. [188] found that Instagram users tend to score higher on body surveillance than non-Instagram users, and greater interaction with image-centric content on Facebook (e.g., updating one’s profile photo and viewing other peoples’ photos) were associated with increased body surveillance. Accordingly, Cohen et al., [190] observed that greater selfie investment (i.e. effort in selecting selfies to post on social media), but not general social media usage, is associated with increased body dissatisfaction and bulimia symptoms. In a sample of adolescents, Wilksch et al., [167] reported that number of social media accounts was positively associated with disordered eating cognition (i.e. concern about weight, shape or eating) and behaviors (i.e. skipping meals). Increased daily time spent on using Instagram and Snapchat were also associated with greater disordered eating behaviours, but only in girls [167]. Facebook users reported significantly lower body satisfaction than non-users [161] and increased emotional connection and integration of Facebook into one’s life was associated with increased online physical appearance comparison, which in turn has been linked with greater levels of disordered eating [113]. Furthermore, viewing idealized and edited Instagram images has been associated with greater body dissatisfaction [181]. Instagram photo-based activities positively predicted both drive for thinness and body dissatisfaction through the mediating variable of appearance-related comparisons [160]. The frequency of Facebook-photo related activities was also positively correlated with internalization of the thin ideal, self-objectification, and drive for thinness, and negatively correlated with weight satisfaction [163].

Tiggemann and Slater [189] found that numbers of Facebook friends, but not Facebook usage, prospectively predicted greater drive for thinness in a sample of adolescent girls, suggesting that it may be the excessive virtual body comparison, but not general social media use, that is driving symptoms of eating pathology. Indeed, among Facebook users, number of Facebook friends was significantly correlated with internalization of thinness ideal, body surveillance, and drive for thinness scores [165].

Kim and Chock [187] reported that it was viewing and commenting on peers’ profiles, but not time spent on social media, that was associated with greater drive for thinness in a sample of young adults. Likewise, Cohen et al., [190] found that greater selfie investment (i.e. effort in selecting selfies to post online), but not general social media usage, was associated with increased body dissatisfaction and bulimia symptoms in women. Moreover, Instagram photo-based activities positively predicted both drive for thinness and body dissatisfaction through the mediating variable of appearance-related comparisons [160]. Indeed, frequency of comparing one’s physical appearance to people followed on social media was associated with greater body dissatisfaction and increased drive for thinness [179]. Notably, one study found that discrepancies between women’s perceived actual thinness and what they perceived to be the thinness ideal of women viewed on social networking sites were associated with the greatest level of body dissatisfaction [178]; in other words, women were more like to experience body dissatisfaction when they perceive that other women on social media held a thinness ideal that was considerably different from their actual body shape. In comparison, discrepancies between women’s actual thinness level and what they perceive their real-life female peers held as “ideal” was still associated with body dissatisfaction, but less strongly so [178]. Accordingly, Yao et al., [180] found that body image comparison on social media was positively associated with restrained eating, which was mediated by body shame. In a sample of female undergraduate students, frequency of Facebook usage was positively associated with body dissatisfaction and drive for thinness (which is mediated by both general appearance comparison), as well as with comparison to friends and distant friends, and upward comparison to distant peers and celebrities on social media [168]. In addition, Lonergan et al. [207] found that both avoidance of posting selfies and photo manipulation were associated with greater adjusted odds of meeting criteria for clinical or subclinical anorexia [207]. The same study has also found that online selfie-posting was unrelated to both eating disorder symptom severity and body dissatisfaction. In contrast, Butkowski et al. [185] reported that frequency of Instagram use was not associated with greater body dissatisfaction or drive for thinness. Finally, in a recent meta-analysis, Ioannidis et al. [208] demonstrated that eating disorders, drive for thinness, and dietary restraint were all associated with increased and ‘problematic’ internet use. Taken together, this evidence suggests that social media use may mediate prevalence or severity of eating disorders by promoting an overly idealized but unrealistic points of comparison for its users, particularly those who may already be struggling with self-identities.

As summarized in Supplementary Table 4, many studies have observed a pattern of greater image-based activities and social media usage and increased disordered eating symptoms and body image distortions. For example, young adults who received extremely negative comments in response to personally revealing status updates were more likely to report disordered eating four weeks later [184]. Greater Instagram use has been associated with greater self-objectification; in particular, more frequently viewing of “fitspiration” images on Instagram is associated with greater body image concerns [209]. Selfie-photo modification is also positively associated with rumination about weight, eating, and shape, which in turn has a positive association with disordered eating [210]. Mclean et al. [182] found that girls who regularly share selfies on social media, compared to those who don’t, were more likely to report body dissatisfaction, greater internalization thinness ideal, as well as increased body-related and eating concerns. Likewise, women who post fitspiration images, relative to women who post travel images on Instagram, scored higher on drive for thinness, bulimia symptoms, drive for muscularity and compulsive exercise; furthermore, approximately twice as many women in the fitspiration group were more likely to be at risk for an eating disorder diagnosis than the travel image group [183]. Maladaptive Facebook usage (i.e., negative self-referential activity: e.g., “Reading the status updates of others tends to make me feel down on myself”) predicted increases in bulimic symptoms and number of over-eating episodes four weeks later [176]. By contrast, one study found no significant association. of social media usage with eating and body image problems in a sample of in a sample of high school students [186]. Considered together, the above studies provide evidence that greater social media usage, particularly those involving visual self-presentation (i.e. viewing selfies or “Fitspiration” images) and attendance to social feedback, are associated with greater eating disorder symptoms.

#### Autism spectrum disorders and social media use

Autism is a neurodevelopmental condition characterized by decreased mentalistic cognition [211].

Although considered as a single condition, it has many genetic and environmental causes, and it is notably diverse as regards the sensory, intellectual, cognitive and affective traits displayed by individuals. As noted above, autistic traits are predicted to be associated with decreased social media usage, as they involve reduced social interests and social interactions [212, 213]. Furthermore, when individuals with autistic traits do use social media, it is predicted to be mainly in the context of non-social (i.e., mechanistic) purposes, such as sharing factual information on topics of special interests.

Nine papers met the search criteria for social media usage in autism spectrum disorders. As summarized in Table 6, seven of the nine available studies indicated that individuals with autism tend to use social media less compared to controls. For example, Mazurek et al., [214] found that compared to youths (age 13–17 years old) diagnosed with other disabilities (speech/language impairments, learning disabilities, or intellectual disabilities), youths with autism spent the highest amount of time (as reported by their parents) on non-social electronic media (e.g., TV, videos), despite being the group with the highest rate of having a computer at home. Furthermore, approximately twice as many youths with ASD did not use e-mail or chatrooms, compared to speech/language impairment and learning disability groups [214], which suggests it is not learning or speech impairments that lead to decreased social media usage, but reduced social interest. Likewise, MacMullin et al., [215] found that individuals with autism, compared to neurotypical individuals, spent less time using electronics for social activities (e.g., social media, talking or texting on a cellphone, instant messaging, using chatrooms, and emails) but more time using the Internet, as well as using the Internet on general non-social activities such as creating documents or editing photos. Similarly, Suzuki et al., [216] found that higher autistic traits correlated with decreased friends and participation in chat groups on the messaging app “LINE” and Instagram use, and Begara Iglesias et al., [217] found that 96% of neurotypical individuals and 82% individuals with intellectual disability used social media, but only 65% of individuals with ASD did so. Individuals with ASD were less likely to use WhatsApp and join fewer Whatsapp groups compared to neurotypical individuals [217] and were also less likely to use electronics (e.g., tablets, phones) to talk with friends relative to neurotypical individuals [217]. Compared to neurotypical children, children with ASD spent less time using social media and more time playing video games, playing video games alone, and were less likely to play socially collaborative video games or play video games with other people [218]. Moreover, boys with ASD also used computer-mediated communication (e.g., emails, text messaging, messaging apps, social media sites) less frequently relative to neurotypical controls [219].

**Table 6:** Relationships of social media usage with autistic traits and autism spectrum conditions

References	Methods	Main Findings
Mazurek et al., 2012 [214]	- Parents of the following groups of youths (age 13–17): (1) autism, (2) speech/language impairment, (3) learning disability, (4) intellectual disability, self-reported their children’s screen-based media use. − 920 participants with ASD, 860 participants with speech/language impairments, 880 participants with learning disability, and 850 participants with mental retardation	- Compared to all other groups, the group with ASD spent most of their time on non-social media (ex: TV, video games), despite being the group with the highest rate of having a computer at home − 64.4% of youths with ASD did not use e-mail or chat-rooms, similar to the mental retardation group (64.4%) but twice as high compared to 33.5% of the speech/language impairment group and 34.9% of the learning disability group
MacMullin et al., 2016 [215]	− 172 parents of typically developing children (mean age = 11.72) and 139 parents of children with an ASD diagnosis (mean age = 12.25) report on their children’s electronic usage	- Individuals with ASD spent less time using electronics for social activities (ex: using social networking sites, using chat rooms, texting/messaging/ talking on cellphone, emailing) compared to NT individuals - individuals with ASD spent more time using electronic devices (ex: laptop, desktop, iphone/smartphone, tablet devices) than the NT individuals - individuals with ASD spent more time on the Internet (ex: websurfing, search engine, watching videos, creating webpages, download music files) and general computer activities (ex: creating documents for school, editing images, using photo editing programs) than the NT group
Mazurek and Wenstrup, 2013 [218]	- TV, video game and social media usage were compared between children with ASD (*N* = 202, mean age = 12.1 years) and typically developing siblings (*N* = 179, mean age = 12.5) via parent self-reports.	- Children with ASD spent less time using social media than TD individuals
Durkin et al., 2010 [220]	- Self-report cellphone usage is compared between adolescents with Asperger Syndrome (*N* = 35, 28 Males, 7 females, mean age = 14.2 year) to neurotypical adolescents (*N* = 35, 29 males, 6 females, mean age = 14.4 year)	- The ASD group had lower cell phone access compared to the NT group - Individuals with ASD ranked “phone friends” significantly lower for cellphone use compared to the NT individuals - Individuals with ASD ranked “play games” as a function for cellphone use higher than TD group
Paulus et al., 2020 [219]	- Frequency of computer gaming and ‘computer-mediated communication’ (CMC) (ex: electronic devices for communication purposes, such as Facebook /Instagram/messaging apps) was compared between 62 boys with ASD (mean age = 11.5) and 31 healthy control boys (mean age = 11.5) via parental reports. - Computer gaming and CMC usage were NOT pooled together.	- ASD boys used CMC (e.g., emails, text messaging, social media sites) less frequently than controls - For the ASD group, the top three CMC used were WhatsApp, Skype, and Youtube. The control group reported similar preferences except Youtube was not a top preference
Suzuki et al., 2021 [216]	- Two surveys were administered to two separate groups of participants to investigate the relationships between autistic traits, loneliness, social media and LINE (a messaging app) usage - Survey one: 341 students (100 men, 241 women, mean age = 20.0) at a Japanese university completed questionnaires on their social media usage, Autism Quotient – Japanese version, and the Japanese version of University of California, Los Angeles Loneliness Scale-Version 3 (UCLA-LS3) - Survey two: a total of 388 undergraduate students (145 men, 213 women, mean age = 19.40) completed a LINE use questionnaire (LINE is a popular messaging apps used in Asia), AQ-Japanese version, and the UCLA-LS3	- Numbers of friends and informal groups on LINE were found to have significant negative correlations with both ASD traits and loneliness - Instagram use negatively correlated with loneliness and ASD traits - Low AQ-social skills scores were found to have a positive association with inactive use of LINE (e.g.,. only use LINE when there is a specific reason to do so”).
Begara Iglesias et al., 2019 [217]	- Social media usage and experiences of cyberbullying were compared between three groups: (1) Neurotypical (*n* = 105, 43.8% male, mean age = 15), (2) Individuals diagnosed with ASD (*n* = 31, 74.2% male, mean age = 15), and (3) individuals with intellectual disability (*N* = 45, 60% male, mean age = 19)	− 64.5% of the people with ASD used social media, compared to 96.2% of the neurotypical group and 82.2% of the group with intellectual disability - 76.7% of group with ASD used Whatsapp, compared to 91.1% of the group with intellectual disability and 96.2% of the neurotypical group - Group with ASD had fewer Whatsapp groups compared to the neurotypical group - Compared with the neurotypical group, the ASD group were less likely to use tablets/cellphone/computer to “talk with friends” - The group with ASD was the least likely to use social media to “communicate with friends” compared to the neurotypical group and the group with intellectual disability
Alhujaili et al., 2022 [221]	− 26 adolescents (age 13–18) with ASD (23 male and 3 female) and 24 non-ASD adolescents (3 male, 20 female) completed a questionnaire on their social media usage	-No differences in average time spent on social media daily between ASD group and non-ASD group; however, 35% of adolescents with ASD spend more than five hours per day on social media compared to 18% of non-ASD adolescents -The most preferred social media site for ASD adolescents was Youtube, and Snapchat was preferred in the non-ASD group -About 92% of non-ASD participants reported social interaction as reason for using social media compared only 7.7% of participants with ASD; in contrast, 59% of ASD participants reported entertainment as their purpose of using social media
van der Aa et al., 2016 [222]	− 113 individuals with ASD 55.9% men, mean age = 40.2) and 72 control individuals (38.9% men, mean age = 40.5) completed questionnaires on internet and computer-mediated-communication use, well-being scales (i.e. self-report satisfaction with one’s online and in-person social life), and the Autism Quotient	-Compared to controls, individuals with ASD spend more time on computer-mediated communication -Out of all CMC channels, people with ASD used discussion sites more frequently than controls

As consistent with ‘real-life’ behaviors (where individuals with autism tend engage in decreased social interactions or less socially motivated), individuals with autism preferred to use phones for games rather than to contact intimate others [220]. Notably, although Alhujaili et al. [221] found no significant differences in average time spent on social media daily between an ASD group and a non-ASD control group, 35% of adolescents with ASD spent more than five hours per day on social media, compared to 18% of non-ASD adolescents. However, the same study also found that individuals with autism were more likely to report using social media for entertainment purposes than social interactions [221]. In addition, relative to controls, individuals with ASD were more likely to perceive the isolated context, asynchronous timing, and absence of non-verbal cues of social media platforms as an advantage rather than disadvantage [222]. Given the above findings, the current data supports the hypothesis that individuals with autistic traits may use social media usage less frequently due to decreased social interest, as consistent with the ‘real-life’ behaviors associated with autistic phenotypes.

Finally, Paulus et al. [220] found that boys with autism reported Youtube as one of their top three preferred social media platforms, a preference not expressed by the neurotypical control group. Adolescents with ASD also preferred Youtube, compared to Snapchat in neurotypicals [222]. Given that Youtube has historically lacked socially interactive features such as live chat/streaming and only involves relatively solitary and asynchronous activities such as video uploading/watching, individuals with ASD may prefer Youtube for its relatively isolative functions. However, given that there are only two studies investigating social media platform preferences between the ASD and neurotypical populations, additional research is needed on this question.

Although most studies indicate that social media usage is reduced in individuals with ASD, one study has found that individuals with ASD spent more time on computer mediated communication (email, Twitter, instant messaging, social networking sites, discussion sites/forums, dating sites, games) than neurotypicals [222]. However, in this study, individuals with autism also reported using discussion boards, which tend to be more factually driven and less socially oriented in nature, more than controls [222]. Taken together, the data suggest that when individuals with ASD use social media, it may often be to communicate factual rather than social information. However, more studies are needed to fill the substantive gaps in this area.

## Discussion

**Fig. 2:**
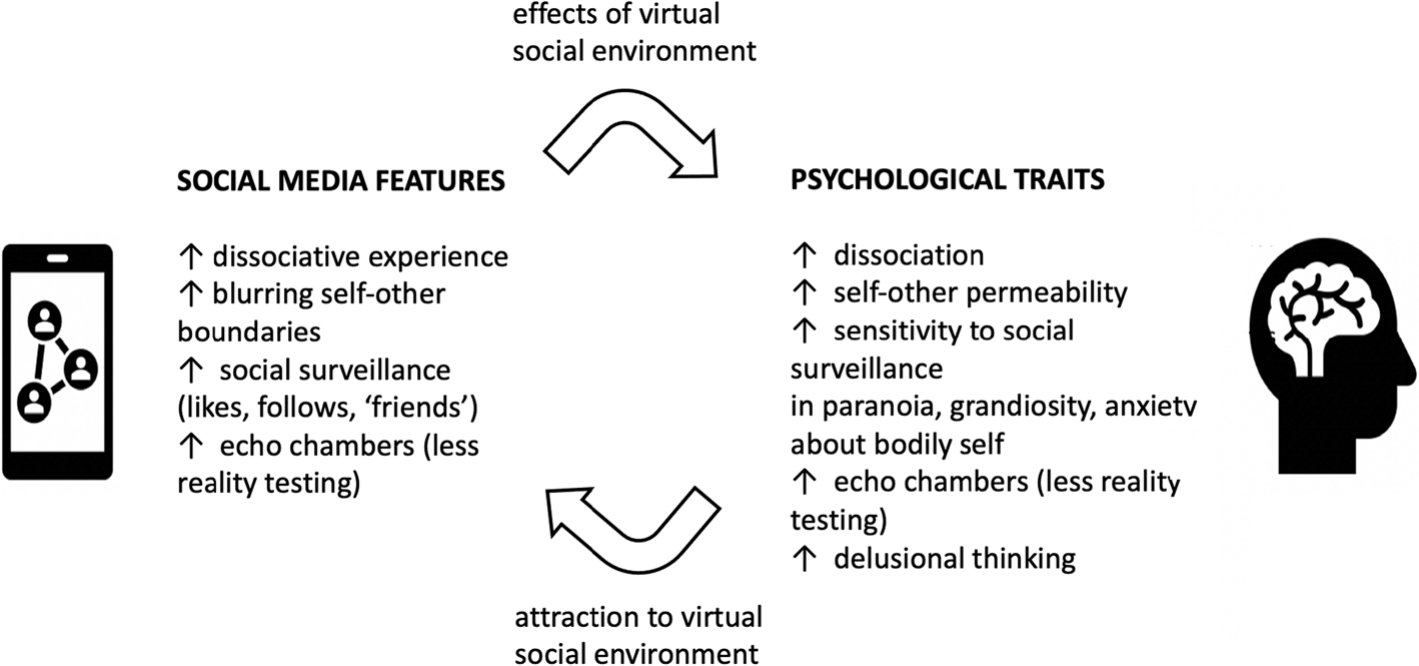
A simplified model of how features of social media may interact with human psychological traits

A list of main findings is summarized in Table 7. The main results, overall, are threefold. First, highly mentalistic traits, including paranoia, erotomania, ideas of reference, and other aspects of positive schizotypy, show diverse evidence of notable associations with relatively high social media use. The causes of these links appear to involve features of virtual social environments, especially those that support imaginative social cognition. These links appear to be bidirectional, in that people with particular traits, especially mentalistic ones, are differentially drawn to social media, and social media, in turn, influences the traits of those individuals due to the features of virtual environments. Thus, (1) more highly mentalistic individuals may be differentially drawn to social media because it provides increased scope and supportive conditions for delusional and distorted thinking while, at the same time, (2) the virtual social environments created by social media platforms may facilitate the expression and exacerbation of social delusions and social-cognitive reality distortions (Fig. 2). This framework goes beyond the view that social media represents a problematic environment that can promote particular forms of mental illness or distress, to suggest that individual psychological makeups interact closely with features of virtual social worlds, especially in the context of high levels of different manifestations of mentalistic cognition. This hypothesis can be evaluated further using controlled experiments, and longitudinal studies, that seek to parse the direction, or directions, of causation.

**Table 7:** Summary of main findings

Disorder or trait	Summary of main findings	Proposed explanation
**Paranoia**	Social media usage involves increased feelings of surveillance and increased self-consciousnesses (i.e. self-surveillance), usually with negative affect	- The openness of online platforms can instill a sense of constant surveillance, as almost anyone anywhere can view, screenshot, record, or download content - Technological advances have broken down traditional privacy barriers (i.e. physical space, time-limited interactions)
**Erotomania**	Social media usage can be associated with onset and severity of erotomaniac delusions, particularly if the individuals were already socially isolated in real life and used social media as substitute for real-life interactions	- Viewing someone’s private life online may create an illusory sense of false emotional intimacy. - Individuals predisposed to ideas of reference may perceive “hidden meaning” or illusory personal reference in general social media posts - Social networks create an illusory sense of available romantic partners, as users can now “connect” to high status individuals
**Psychosis**	Increased social media usage is sometimes associated with onset of psychotic episodes, particularly in ways that reinforced existing delusional beliefs (i.e. conspiratorial thinking, thought insertion, thought broadcasting, being controlled by an external agent)	- Lack of embodied exchanges in online interactions can weaken intra and interoceptive processes that normally underlie reality testing - Hidden website trackers and search algorithms may reinforce delusional beliefs as online content is curated to prior search content
**Schizotypy**	Positive schizotypal traits may be associated with increased social media usage and certain online activities (e.g. online gaming, internet chat rooms, emails). However, one study has found social media usage is reduced among non-clinical individuals with psychotic-like experiences.	- Certain facets of positive schizotypal traits, such as social anxiety, may increase social media usage for its relative anonymous and distancing nature. However, other aspects of positive schizotypy, such as paranoia, may reduce social media use, due to higher social anxiety
**Narcissism**	Increased social media usage is associated with narcissistic traits. Individuals with higher narcissistic traits also tend to use social media in more self-exhibitionist ways	- Collection of likes/online followers/friends amplify pre-existing beliefs of grandiosity - Most social media platforms are exhibitionistic and self-promotional, which aligns with the narcissistic beliefs
**Body dysmorphia/ Anorexia**	Increased social media usage is associated with body dysmorphia and eating disorder symptoms	- Individuals with body dysmorphia and eating disorders have a weak and unstable sense of self and use social media as an external “scaffold” to self-regulate an unstable body image - Social media functions as a virtual social “mirror” that helps to provide 24/7 external “feedback” to body-checking behaviors

Second, the analysis of psychiatric disorders in relation to social media use provides evidence that psychotic spectrum disorders involve relatively high levels of this behavior, although the data are, for some disorders (including schizophrenia, schizotypal personality disorder, and bipolar disorder), sparse, especially given the prominent roles that social media and internet use play in contemporary society. Despite these limitations, there is notable evidence that high social media usage is consistently relatively-strongly associated with a small set of disorders that involve psychotic traits (especially delusions): narcissistic personality disorder (NPD), body dysmorphic disorder (BDD), and eating disorders. Why should this be so?

**Fig. 3:**
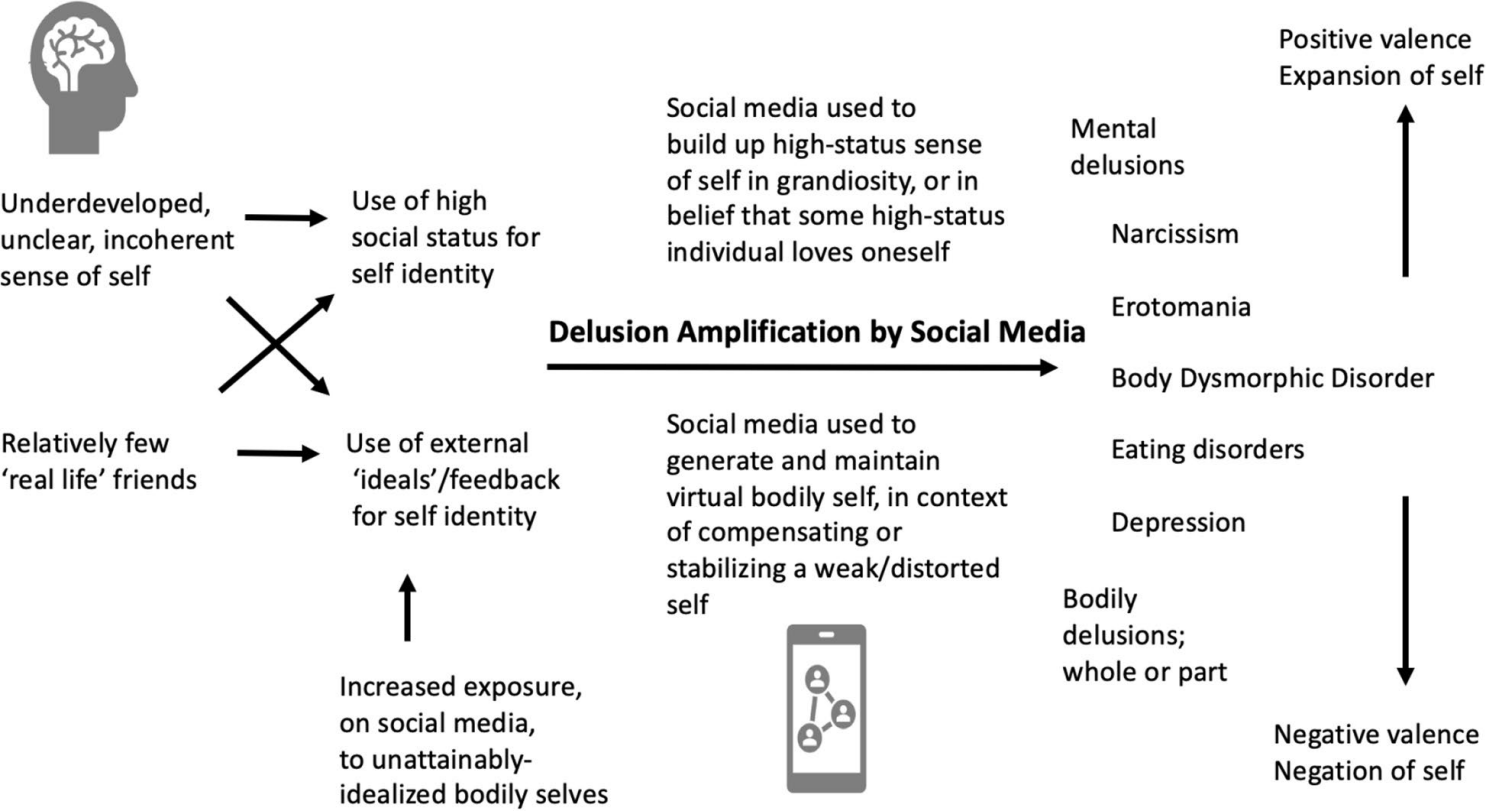
The delusion amplification by social media model, in explaining high social media usage in self disorders

We suggest a simple framework, that we refer to as the Delusion Amplification by Social Media (DASM) model, whereby NPD, BDD, eating disorders, and apparently erotomania, all centrally involve forms of mentalistic delusions, linked with altered perception and perpetuation of distorted manifestations of the self, that are specifically and especially enabled and exacerbated by social media, though in different ways with different emotional valences (Fig. 3). In particular, by this model, an underdeveloped and less-coherent sense of self, in conjunction with ‘real life’ social isolation that inhibits identify formation and facilitates virtual social interactions, leads to use of social media to generate and maintain a more or less delusional sense of self identity. The delusions involved may be mental (as in narcissism and erotomania), or somatic (as in BDD and eating disorders, encompassing either the entire body or specific parts). In each case, the virtual nature of social media facilitates the delusions because the self can be mentally defined and bolstered in this highly mentalistic environment, where face to face scrutiny, and potential real-life exposure of the delusion, are largely avoided.

The specifics of the DASM model depend on the disorder. Thus, in individuals with NPD, the self becomes over-expanded and grandiose in terms of gaining high status [223] (sometimes in association with underlying mental vulnerabilities) and exaggerated positive self-perception (i.e., positive self-illusion) [224, 225]. Indeed, narcissistic traits have been associated with a greater likelihood of perceiving social “others” as extension of oneself, and a greater likelihood of objectifying others for self-gratification [226]. Accordingly, narcissistic individuals tend to seek romantic partners for their self-enhancement value instead of relationship quality (i.e., as “trophy” spouse vs. emotional intimacy [227, 228], and choosing friends for self-enhancement purpose (ex: status, attractiveness, “makes me feel good”) [229], all of which reflect tendencies of perceiving social “others” as instrumental objects for self-gratification, rather than as separate individuals with their own feelings, beliefs, or wants. In addition, narcissism is associated with greater exhibitionistic behaviors on social media, such as increased status updates, selfies, and more online friends/followers, which is consistent with the behaviors of an overly idealized self-presented to a positive and widespread social gaze. Considered together, these findings suggest that the perception of social connection and surveillance, the main functions of most social media platforms, serves to amplify and mirror back an over-idealized self to the narcissistic individual, which further increases an inflated self-perception that incentivizes increased social media use.

In contrast to NPD, BDD and eating disorders are usually associated with negative affect, anxiety, distress, and high levels of body dissatisfaction. If increased social media usage involves increased anxiety and distress among the individuals with BDD or eating disorders, what would explain their increased usage?

BDD and eating disorders have been characterized as involving distorted or reduced sense of self, with weakened self-other boundaries, diminished autonomy, and high levels of self-body alienation [230–234]. The body is thus commonly perceived and instantiated as an external object that is seen through the eyes of the social “other”, rather than being experienced through lived corporality (i.e., via basic self through first-person point of view) [234, 235]. This altered perception may cause anxiety from feelings of being scrutinized, but also inadvertently help to define and maintain the basic self-identity that, though distorted, is required for self-coherence and interpersonal functioning. Indeed, some authors have speculated that distorted body image in individuals with eating disorders may stem from a weaker experiential self, and thus such individuals may be more motivated to seek external and “objective” parameters to instantiate their identity, such as weight (i.e., a numerical value), a mirror (i.e., to check one’s reflection), or the gaze of others (i.e., excessive preoccupation of others’ opinions) [236]. Regarding the latter, the advent of social media provides a concrete and quantifiable way of objectifying and validating the external social gaze, via “likes”, “comments”, number of views, and follower or friend counts. Thus, affected individuals may turn to social media usage as a way of personifying and stabilizing a sense of embodied self by attending to external signals of simulated embodiment, for which social media platforms have specific, curated, online tools.

Importantly, the virtual nature of social media also means that it can also be used in attempts to create and perpetuate a bodily self lacking in perceived flaws. However, given that the platforms lack the critical component of providing non-mentalistic, real-life perceptual physical stimuli, increased usage may exacerbate, rather than stabilize, increasingly distorted self-images, leading to increased dysmorphia and eating disorder symptoms rather than their mitigation.

NPD, BDD, eating disorders, and erotomania thus appear to share a set of key developmental risk factors, involving formation of an underdeveloped or incoherent sense of self, and be similarly facilitated by social media use, through its ability to perpetuate mental or somatic delusions that sustain the altered structure of self-identity. The specific forms and demographics of these delusions can also be contextualized in the framework of human life histories, in that (1) male reproductive success value as mates are enhanced by high social status [237–239] which is a central focus of narcissism, whereas female reproductive success is associated with physical beauty [240–242], as well as having a high-status mate [243]. In accordance with these findings from studies of human behavior and reproduction, NPD is highly male-biased [244], eating disorders and erotomania are highly female-biased [245, 246], and BDD, which involves beauty-related traits in females but muscle-related traits in males [247] (which are also of high value in mate attraction, which also shows a relatively unbiased sex ratio [248].

NPD, BDD, eating disorders, and erotomania, also entail forms of delusionality, and thus psychosis-associated traits, although their symptoms and intensity are highly variable across individuals. They can, however, be conceptualized along a spectrum that, at its extreme, involves high levels of self-other permeability [249], literal ‘dis-integration’ of the basic self, and first-rank psychotic symptoms whereby the basic experiential sense of agency and body ownership are externalized and projected outward [250].

The above considerations in no way deny the roles of social media in directly exacerbating BDD and eating disorders through evolutionarily-novel, culture-based effects on striving for unrealistic bodily ideals of thinness or beauty (e.g., [251]). Instead, they provide simple, testable models for helping to explain why social media use is so strongly associated with this small, specific set of psychiatric traits and conditions.

Although depression has not been addressed in any detail in this review, it can also be considered in the context of the DASM model, given the large body of literature on the relationship between increased social media usage and this condition (e.g., [252–255]). Although depression is normally conceptualized as a mood disorder, recent evidence suggests that it may also be considered as a disorder involving embodiment [256, 257]. As with other conditions of bodily delusions (e.g., body dysmorphia, eating disorders), patients with depression experience symptoms suggestive of a diminished or negated sense of self (i.e., feeling of unworthiness, emptiness, social withdrawal, low self-worth) [258], or an overly porous self-other boundary where the individual with depression tend to over-identify with another person’s distress (i.e., excessive “empathy”) and consequently experience inappropriate self-blame [259]. Indeed, depression is also characterized by excessive mentalizing emotions such as guilt and shame [260, 261], both of which involve simulating and internalizing the mental state of the social “other” in self-evaluations [262, 263]. Taken together, these considerations suggest that, as with other self-delusions (e.g., body dysmorphia, eating disorders), depression may also be associated with increased social media usage via its other-centered cognitive orientation [259], which manifests itself, in part, in terms of maladaptive upward virtual social comparison [264].

Although evidence for effects of social media and internet use on liability to psychosis per se (as in schizophrenia or bipolar disorder) comes only from case reports, rather than from quantitative analyses, multiple congruent reports of internet or social media-related psychosis have been reported in schizophrenia patients who were spending extreme amounts of time online in lieu of real-life social interactions, with psychotic symptoms involving ideas of reference in online content, magical thinking, and loss of body or thought ownership or agency. These reports suggest a degree of enmeshment between the self vs. non-self in virtual mental spaces, where basic thought, agency, and body ownership are externalized to virtual others. In this context, social media can be seen as presenting an evolutionarily novel medium where there is considerable blurring and ambiguity of self-other (i.e., private-public) boundaries, and where much activity is “other-centered” and externally directed (i.e., social surveillance, receiving likes/follows/” friends”, curation of photos and online content to be viewed or consumed by an imaginary audience) – all of which may predispose an individual with a more porous sense of self to reductions in their sense of agency or body/thought ownership.

**Fig. 4:**
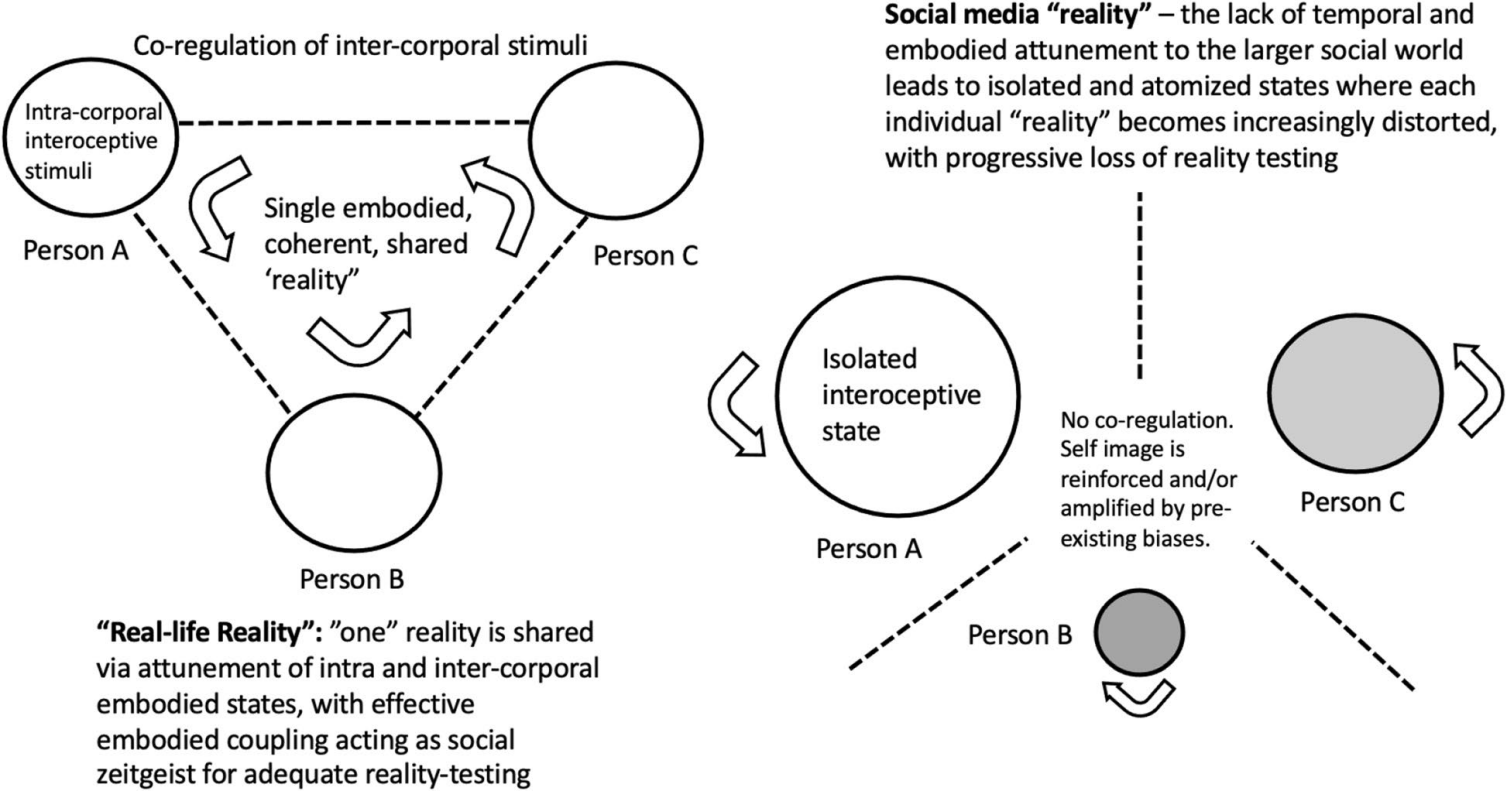
A model for how social media alters the nature of realities in the context of self-other interactions

How might social media platforms increase the prevalence or severity of self-disorders, at least in principle? In conjunction with the DASM model, we propose a hypothetical framework for how disembodied social interactions in virtual spaces may lead to failures in reality testing due to loss of inter-corporal coupling (Fig. 4). As described above, humans have evolved to socially interact with one another in shared physical and temporal spaces where there is a “to-and-fro” of synchronization and de-synchronization for various intra-corporal states (i.e., facial expressions, gestures, emotions) and inter-corporeal states (i.e., mirroring of body language, tone, pace of speech). For example, Fuchs and De Jaegher [265] observed that it is the dynamic oscillations between embodied attunement (e.g., mirrored body language, emotional co-regulation) and alienation (e.g., conflict, disagreement) that allows social agents to successfully interact with one another in individuated states. By this view, the lack of desynchronization, or “perfect synchronization”, of algorithm-driven social media feeds into its individual user’s activities and preferences and may lead to a collapsed “undifferentiated, homogeneous feeling state” ( [265] pp. 471), or the loss of self-other boundary [266], manifesting in the type of psychotic symptoms summarized in Tables 3 and 4.

This perspective on disorders of the self, in relation to social cognition, is predicated on a view of social cognition and its development as embodied, enactivist and based on interactions, coordination, and shared mental representations between individuals [256, 263, 265, 267]. The primary empirical evidence for this perspective, in comparison to ‘representationalist’ viewpoints based on inference or modelling of the mental states of others (e.g., Deschrijver and Palmer [268] comes mainly from studies showing that bodily states affect cognitive process, that sensory and motor areas become active during cognition, and that mirror neuron activation serves as a core basis for social interaction, coordination and understanding (e.g., [265, 267, 269]).

In the context of the framework in Fig. 2, clinical reports of internet or social-media related psychosis commonly follow a pattern that starts with real-life social isolation (decoupling of self from one’s physical social world), increasing usage of online social activities in lieu of real-life social interactions, and prodromal symptoms such as ideas of reference and magical thinking. Then, in high-risk individuals, full blown psychotic beliefs may develop, where the patient experiences a loss of boundaries between self (internal) or non-self (external) environment, leading to a variety of delusions such as erotomania, paranoia, being controlled/followed, and conspiratorial plots [20, 21, 37, 38, 43, 45], all of which involve a blurring of self-other boundaries, which is common in individuals with psychosis or schizophrenia [266, 268]. Furthermore, unlike real-life exchanges, online social interactions lack the physical component of shared inter-corporal co-regulation, which may lead to a positive feedback loop whereby an individual increasingly turns to the online world (in lieu of the real one) for reality-testing but becomes increasingly distressed and entrenched in pre-conceived distorted self-images due to lack of enactivistically embodied stimuli that would normally co-regulate in-person exchanges (Fig. 4). In line with this view, individuals with body dysmorphia or disordered eating symptoms tend to experience greater symptom severity with increased usage, as described above. Again, additional quantitative data is needed to evaluate this model of how increased social media usage may lead to disorders of disembodiment.

The third main finding of this study is that individuals on the autism spectrum show suggestive, though mixed, evidence of reduced social media use. Individuals on the autism spectrum thus show evidence of being less ‘other’ and more ‘self’ oriented, with stronger self-other boundaries [233, 270], which may reflect a patterns of decreased social media usage overall [214–219, 221], as well as preferences for the relatively historically solitary platform Youtube [219] instead of Instagram [216] or Snapchat [221]. Furthermore, given that Youtube use is relatively more male-biased [271] than other internet platforms, and has historically involved content focused on special interests, the finding of individuals with autism preferring Youtube over other forms of social media may be another indicator of less mentalistic cognition among individuals on the autism spectrum. In this context, it is also important to note that autism commonly involves high levels of sensory sensitivity that can themselves interfere with social interaction, through sensory overload, challenges with multisensory integration, and the creation of problems with embodiment serving as a mechanism for social connectedness (see [272–274]).

Several lines of evidence also suggest that individuals with ASD utilize social media for more non-social purposes and to avoid difficulties associated with interpreting non-verbal cues in face-to-face exchanges. For example, individuals with ASD have consistently reported preferences for using text-based, asynchronous communication over “traditional” ones, as it allows for pacing of responses, minimized non-verbal cues [275–277] (Massier LA: Computer-mediated communication usage and perceptions amongst young adults with autism spectrum disorder, unpublished) and a sense of interpersonal detachment (i.e., “…can connect with others while maintaining a level of detachment…not worry about physical or in-conversation cues” “…communicate with people …in a format that I am comfortable with – limited emotion – no dumb small talk”) [278]. Taken together, decreased social media usage in individuals on the autism spectrum disorder may reflect decreased interest in social interactions in general, which may be considered a subcomponent of mentalistic cognition.

Finally, given that social media platforms are being increasingly incorporated into everyday life, the increasing enmeshment between online vs. offline activities may create novel cognitive pressure toward “extended digital phenotypes”; that is, the sense of one’s interpersonal mental and perceptual space being increasingly expanded to include virtual social activities. In this light, the models in Figs. 1, 2 and 3 represent different manifestations of interactive positive feedback loops of the evolutionarily novel digital social migration. In other words, the bidirectional relationship between users’ mentalistic traits and social media platforms’ immersive functions create a vicious cycle whereby the environment selects for higher expression of mentalistic socio-cognitive phenotypes characterized by weaker self-other boundaries, and vice versa. Individuals with a more porous basic self may thus gravitate toward social media platforms for their sense of identity, generating positive feedback loops where pre-existing biases or beliefs are entrenched or amplified due to activity patterns of self-attunement. As more and more social activities become virtualized, fractured and atomized, social media virtual “realities” may emerge as the predominant cognitive ecosystem where each individuals’ social “reality” becomes more and more decoupled and isolated from one another. The decoupling of each user’s social “reality” may in turn lead to progressive loss of embodied and physical reality testing that humans have evolved with, which may exacerbate or amplify conditions characterized by weak self-other boundaries.

Some researchers have suggested that contrary to the viewpoints and interpretations described here, online interactions can still be effective for social-cognitive interaction through their ability to generate ‘felt togetherness’ or ‘we-experiences’ that involve sharing of common awareness, intentions and experiences (e.g., [279, 280]), and given that they can, to some degree, elicit and represent embodiment. We agree that online interactions can involve meaningful cognitive-affective interactions, that include some degree of embodiment, but our main focus and emphasis is that there are profound differences between online and in-person social interactions and environments, that can potentiate and exacerbate deviations from mental well-being. Key empirical evidence for this statement comes from experimental studies that compared aspects of brain activation between individuals engaged in (otherwise-matched) online versus in-person social interactions. Thus, (1) Karimova at al. [281, 282] showed that mirror neuron system activation was substantially greater for individuals in personal direct interactions, than for individuals interacting online, and (2) Zhao et al. [283]) demonstrated that real faces differed notably from onscreen, zoomlike faces for a range of measures including eye-tracking, pupillometry, EEG, and near infrared spectroscopy. Comparable results apply from studies of mirror neurons in macaque monkeys [284], analyses of the mirror neuron system using 3D versus 2D images [285], and studies of mirror neuron system activation in real-word vs. video setting, where the latter completely lacked anticipatory mu suppression activity (Krol and Jellem [286]).

Considered together, these studies indicate that online and in-person interactions show large, clear differences in neural processing for social-cognition paradigms. Such differences may help to explain many of the gaps and distortions that typify the social media interactions described here, as well as phenomenon such as ‘zoom fatigue’, which can be characterized as some function of awkward turn-taking, lack of eye contact, restricted mobility, inhibited spontaneity, and increased self-awareness due to the viewing of oneself [287].

We also suggest that some of the “felt togetherness” of online exchanges may be due to the unidirectional projections of hyper-mentalized cognition. As such, disembodied social interactions may distort and degrade reality testing that is normally embedded within reciprocated and embodied interactions. The atomized nature of “felt togetherness” or “we-ness” of online exchanges could thus tend to exacerbate delusions and psychotic symptoms, rather than ameliorating them.

## Limitations

The findings from this review are constrained by several limitations. First, levels and patterns of social media usage in some disorders, including autism, schizophrenia, schizotypal personality, bipolar I disorder, and anorexia, have been subject to relatively little quantitative study, which limits the strengths of inferences in this regard.

Second, although multiple case reports have focused on individuals with psychosis whose manifestations centre on the internet or social media, quantitative research is needed to elucidate their relationships and evaluate underlying mechanisms. Currently, only case reports are available on the potential relationships between psychosis and social media usage, which makes it difficult to discern whether such casual relationships exist, or if psychotic symptoms were already present prior to increased internet and social media usage.

Third, although most studies examine social media usage as a single construct, certain psychotic spectrum conditions may opt for certain social media functions more frequently than others (for example, narcissism and selfie-taking), and for very different purposes (i.e., admiration or attention seeking in narcissism, body checking in BDD). Future research may usefully consider investigating how usage of different social media platforms (such as image-based Instagram vs. text-based Reddit) and their respective functions (such as selfie or passive scrolling) differ across individuals with different psychological makeups.

Fourth, recent studies of social media usage and eating disorders sometimes investigate the latter as a single relatively homogenous construct, which is likely problematic with regard to discerning their links with social media use. As such, although current data in this review point to relationships of social media usage patterns with distorted body images, and eating disorder symptoms, additional data are needed to evaluate these links in relation to specific eating disorder symptoms, and to see if patterns can be extrapolated to clinical populations, particularly in the case of anorexia.

Finally, although the current research paradigm has predominately focused on cultural explanations (i.e., unrealistic beauty standards) for elucidating the relationships between social media, body dysmorphia, and eating disorders, current data indicates that they may also conceptualized as disorders of embodiment, of which the body image is a symptom, rather than the cause, of pathological eating patterns or altered somatosensory perception.

## Conclusions

The studies and conceptual frameworks described here suggest that social media use is associated with higher levels of mentalistic cognition in general, and with specific mentalistic disorders characterized by symptoms of perturbed embodiment, and we suggest reasons why this may be so. These results in general, and the DASM model in particular, motivate a number of suggestions for applications, as well as recommendations for future studies. First, these findings suggest that individuals with disorders and traits that involve high levels of delusionality (i.e., narcissism, body dysmorphic, anorexia, paranoia, psychosis) would especially benefit from reducing their social media use, to help alleviate the negative impacts of this activity, as well as from developing awareness of the problematic features of social media platforms that are salient to their particular disorder and situation. Second, studies are needed that help to determine the specific, mentally-manipulative (e.g., White et al. [288]) features of social media platforms that encourage and sustain delusions and paranoia, using experiments as well as other methods. Second, the DASM model can be evaluated by testing its prediction that altered sense of the self, combined with a general lack of real-life friends, promotes the use of social media in different ways and for different purposes, depending upon the disorder (Fig. 2). Third, further work is needed to identify the presence and patterns of differences in neuropsychological and endocrine traits between online interactions versus in-person interaction, including study of the differences between communication by text only, audio only, video plus audio, and in-person interaction in pairs or groups; these studies can also usefully determine degrees of hyper-mentalizing and hypo-mentalizing associated with these different conditions. The results of such studies can be used to direct additional research on ways to make online social interactions more embodied, socially grounded, shared, and interactive, though use of eye-contact technology, 3D perspectives, avatars, and other means (e.g., Tan et al. [289]).

According to Cooley’s [290] theory of the looking-glass self, people’s self-identities are formed in three steps: (1) people imagining how they appear to other people, (2) people imagining how others are judging them based on appearance and how they present themselves, and (3) people imagining how others feel about them based on the judgements they make. Considering modern technological advances, the reflecting power of another person’s eyes is being replaced by the glow of smartphone screens displaying virtual faces and bodies. For the first time in human evolutionary history, social interactions can thus be completely disembodied and dissociated from its physical, temporal, and tactile cues.

Such targeted studies, designed to test theories and directionalities of causation, are required to better understand the societal and psychological effects of our novel, virtual, looking-glass world.

## Supplementary Information

Supplementary Material 1.

Supplementary Material 2.

Supplementary Material 3.

Supplementary Material 4.

## Data Availability

The datasets used and/or analyzed during the current study are available from the corresponding author on reasonable request.

## Abbreviations

NPD: Narcissistic Personality Disorder
BDD: Body dysmorphic disorder
ASD: Autism Spectrum Disorder(s)
DASM: Delusion Amplification by Social Media model
BPD: Borderline Personality Disorder
HPD: Histrionic Personality Disorder
