# Supplementary material for: I tweet, therefore I am: a systematic review on social media use and disorders of the social brain
Source: BMC Psychiatry. 2025 Feb 3;25:95. doi: 10.1186/s12888-025-06528-6 (PMC11792667; doi:10.1186/s12888-025-06528-6)
Supplement: Supplementary file 1 — Supplementary Material 1. [file 12888_2025_6528_MOESM1_ESM.docx]

**Supplementary Table 1.** Case studies of social media usage associated with erotomaniac delusions

| Reference | Case | Age | Sex | Psychiatric history | Case reports |
| --- | --- | --- | --- | --- | --- |
| Sayar and Senkal, 2014 [21] | 1 | 40 | F | Depression | Patient lived alone and was unemployed when she was first brought to medical attention. Patient fell in love with someone she “met” on Facebook and persisted in sending the “friend” symbolic messages of their love despite the person having cut off all contact. |
|  | 2 | 35 | F | None reported | Patient was single, lived alone, and spent majority of her free time on social media. Patient fell in love with a man she “met” on Facebook and was devastated when he rejected her advances. However, the patient remained fixated about their “love” and consistently spoke of the man being “inside of her”. |
|  | 3 | 20 | F | Depression with psychotic features | Patient reportedly spent entire days surfing on Facebook. Patient reportedly fell in love with a man on Facebook fell in a psychotic depression when he rejected her in person. |
| Faden et al., 2017 [37] | 1 | 24 | M | None reported | Male college student developed the erotomaniac delusion on a female classmate and started stalking her on Twitter and in-person. Patient was also convinced that multiple other women were in love with him despite lack of evidence. |
| Kuo and Hwu, 2007 [35] | 1 | 18 | F | None reported | The female patient fell in love with a man that she has only “met” online. Patient had communicated with the man over email for three years. Patient became suicidally depressed after receiving a message from another online friend that the “boyfriend” and his family had killed themselves. Patient was convinced that she was to blame for the suicides and became suicidally depressed. |
| Krishna et al., 2013 [22] | 1 | 21 | M | Had seen a counselor for “depression” in high school. Patient’s mother and half-sister had depression. | Family described the patient as introverted and isolative. Patient led a socially isolated life where he worked a job with minimal social interactions and only interacted with “friends” via Facebook. Patient developed an erotomaniac delusion on a female classmate who had accepted his online Facebook “friend” request. Although they never communicated offline, the patient started over-identifying with the female classmate and interpreted her social media updates as evidence of her “love” for him. |
| Prasad et al., 2020 [36] | 1 | 21 | F | None | Patient developed an erotomaniac delusion that she was in love with a boy from her 12^th^ grade cohort. Patient would message men with similar sounding names on Facebook and send inappropriate messages to them. Patient is noted to be constantly messaging men on Facebook, even during the ward stay when she was hospitalized. |
