# Supplementary material for: I tweet, therefore I am: a systematic review on social media use and disorders of the social brain
Source: BMC Psychiatry. 2025 Feb 3;25:95. doi: 10.1186/s12888-025-06528-6 (PMC11792667; doi:10.1186/s12888-025-06528-6)
Supplement: Supplementary file 2 — Supplementary Material 2. [file 12888_2025_6528_MOESM2_ESM.docx]

**Supplementary Table 2**. Reports of psychosis associated with social media or internet usage, or specific mentalistic aspects of social media systems.

| Reference | Case | Age | Sex | Psychiatric history | Case reports |
| --- | --- | --- | --- | --- | --- |
| Kalbitzer et al., 2014 [38] | 1 | 31 | F | None reported | Patient was using Twitter excessively approximately one year prior to psychiatric admission, neglecting her personal relationships for tweeting several hours a day. Patient suffered from paranoid delusions and perceived special hidden meanings in her retweets and felt compelled to carry out special “tasks” as a result (ex: picking up a piece of tissue when she heard a car honk) |
| Lerner et al. 2006 [46] | Case 1 (Ms. B) | 24 | F | None reported | Patient lived alone without family or social support. Patient was reported to be fearful, shy, and rarely went out with friends. Patient developed the psychotic belief that she was being “irradiated” by the internet and that anyone who had access to a computer was able to connect with her, monitor her, and control her thoughts and actions. |
|  | Case 1  (Mr. C) | 28 | M | None reported | Patient was a single man and appeared 'cold and aloof'. As an adolescent the patient spent hours in his room programming and rarely went out with his classmates. Patient wore a saucepan to protect himself from the Internet and believed that there was a “world energy” from the Internet that affected his mind and body. Patient also believed that there was a minicomputer in his head. |
| Bell et al. 2005 [39] | Case 1 | 31 | F | Previous diagnosis of bipolar disorder | Patient believed that she had uncovered secret information about the Al-Qaeda terrorist online and, consequently, believed that her communications were tapped. Patient believed that she was bugged with secret microphones and camera. |
|  | Case 2 | 42 | M | None, but had consulted GP previously for low mood and suspiciousness | Patient believed that websites of international companies were being used by a “secret organization”, which was out to persecute him. Patient believed that indecent images of his wife and daughter were being distributed online by the secret organization. |
|  | Case 3 | 36 | F | Bipolar disorder | Patient believed house was bugged by cameras that were monitoring and broadcasting her activity online |
|  | Case 4 | 19 | M | Schizophrenia, substance and alcohol abuse | Patient believed that he was followed by “thirty to forty people”, who were disgusted with his past misdeeds and wanted him back in the hospital (patient was previously hospitalized). Also believed that the internet was being used to broadcast his past offences (i.e., a practical joke he had done in the past) |
| Schmid-Siegel et al. 2004 [49] | Case 1 | 36 | F | Paranoid schizophrenia | Patient believed that her activity was being broadcasted online. Also believed that chip was implanted in her brain and used to broadcast everything she was seeing online |
| Margolese et al. 2002 [40] | Case 1 | 26 | M | Schizophrenia | Patient believed that people were following his activities online, and that several websites were dedicated to him based on similarities between the websites’ names and his first name. Patient also experienced paranoid ideas of reference from TV and radio. |
| Kobayashi et al. 2001 [50] | Case 1 | 57 | F | Schizoaffective disorder | Patient heard commanding hallucinations through the internet. |
| Podoll et al. 2000 [41] | Case 1 | 32 | M | None reported | Patient believed that he was receiving messages over the internet, which threatened to expose his use of internet pornography. |
|  | Case 2 | 19 | M | None reported | Patient believed that Bill Gates was destroying his files, as well as stalking him online. Also believed that his personal files were duplicating themselves and broadcasted online. |
| Catalano et al., 1999 [47] | Case 1 | 40 | M | None reported | Patient believed that friend was posting erotic videos of him on the internet. Patient also believed that he was being bugged by a friend who worked in the CIA. Also believed that his body was connected and controlled by the internet via weblinks. |
|  | Case 2 | 41 | M | None reported | Patient identified as a witch and believed that he could surf the internet with his mind. Also believed that he was receiving “magnetism” from the internet. |
| Tan et al., 1997 [42] | Case 1 | 27 | M | Body dysmorphic disorder | Patient believed that the internet was controlling his life. Believed that neighbour was broadcasting his life online. Proposed to a woman that he had connected online and then believed that they were plotting to kill him. |
| Duggal et al., 2002 [43] | Case 1 | 31 | M | Paranoid schizophrenia | Patient believed that he was being persecuted by his sister-in-law, who was controlling and monitoring his thoughts through the internet |
| Compton, 2003 [48] | Case 1 | 53 | F | “Previous hospitalization” not specified | Patient believed that the internet was controlling her movements and home appliances. |
|  | Case 2 | 21 | F | None reported | Patient believed that microchips were implanted in her body/clothes to track her behavior. Also believed that her life was being broadcasted online |
|  | Case 3 | 64 | F | None reported | Patient believed that the “www” people (referring to the world wide web) were following and monitoring her whereabouts by tapping her apartments. |
| Nitzan et al., 2011 [44] | Case 1 | 45 | F | None reported | Patient reported that she had turned to social media sites to relieve loneliness after her client (patient was a caregiver for an old man) has died. Patient reported that her online interactions felt disorienting (“You don’t see a human being in front of you… I didn’t know who I was communicating with and who was communicating with me…) and experienced ideas of reference using social media and developed paranoia that other online users were after her |
|  | Case 2 | 30 | F | Previous history of anxiety | Patient started communicating with a man via Facebook and developed ideas of reference from his status updates, interpreting special meaning hidden in the clips/colours/words that the man posted. Patient reported that communicating with the man via Facebook took up most of her day. Over time, patient developed paranoid delusions about the man as she could no longer trust that their messages were private, and that the man and his family might harm her. |
|  | Case 3 | 30 | F | None reported | Patient developed intimate feelings for a man that she has met online and started experiencing tactile hallucinations as their “relationship” progressed (“I actually felt his hand touching me… on my stomach”) |
| DiCarlo and Ghaemi, 2020 [45] | Case 1 | 43 | F | Prior history of postpartum depression at 31 and ADHD | Mrs. A started developing delusional beliefs  after her cellphone was hacked. Patient was convinced that her TV was hacked and stayed up late at night researching hacking, talking excessively about her hacking concerns, and searched the house for extra wires. Patient later developed full-blown mania episodes with hacking delusions. |
