# Supplementary material for: I tweet, therefore I am: a systematic review on social media use and disorders of the social brain
Source: BMC Psychiatry. 2025 Feb 3;25:95. doi: 10.1186/s12888-025-06528-6 (PMC11792667; doi:10.1186/s12888-025-06528-6)
Supplement: Supplementary file 3 — Supplementary Material 3. [file 12888_2025_6528_MOESM3_ESM.docx]

**Supplementary Table 3.** Evidence relevant to association of narcissism with social media usage

| References | Method | Main findings |
| --- | --- | --- |
| Bergman et al., 2011 [136] | - 361 undergraduate students born in the Millennial generation (mean age = 20.8, 53.6% male) completed a questionnaire on social media usage and the Narcissistic Personality Inventory | - Narcissism did not predict amount of time spend on social media, frequency of status updates, posting picture of others, or checking up on social media friends  - Narcissism predicted the following motivation for using social media: (1) getting as many social media “friends” as possible, (2) wanting social media friends to know what they were doing, (3) believing that their social media “friends” were interested in what they are doing (4) projecting a positive image with their social media profile |
| Mehdizadeh, 2010 [77] | -100 undergraduate students who were Facebook users (50 male, 50 female; mean age = 22.2) had their Facebook page rated by the researcher. Participants were also administered a survey on demographics, Facebook activity, the Rosenberg Self-Esteem Scale, and Narcissism Personality Inventory NPI-16 | - Higher narcissism predicted number of times Facebook was checked per day, time spent on Facebook per session, and greater self-promotion behaviors on the Facebook photos, status updates, notes, but not self-promotional behaviours on the “About Me” section on Facebook |
| Panek et al., 2013 [78] | - Study one: 486 undergraduate students (74% female, mean age = 18.8) that completed an online survey on social media use and attitudes, and the 16-item Narcissism Personality Inventory (NPI-16)  - Study two: 93 adults recruited from MTURK (58% female, mean age = 35.2) completed a survey on social media use and the 40-Item Narcissism Personality Inventory | -Narcissism predicted Facebook status updates, daily use of Facebook, and Twitter posts but not daily Twitter use in the sample of college students  - Narcissism predicts frequency of Facebook posting in the adult sample |
| Poon and Leung, 2011 [97] | - Snowball sampling of 344 Internet users, aged 13-33 years old (47.4% male, 67.7% female) completed questionnaires on “gratification” reasons for producing online content (ex: needs for recognition, venting negative feelings), short version of the Leisure boredom Scale, Narcissistic Personality Inventory, self-reported levels of online content production | - Narcissism predicted greater production of online content on social media sites, blogs, personal webpages, and forums |
| Skues et al. 2012 [137] | - 393 first year undergraduate students (300 women, 93 men, mean age = 20.6) from an Australia university completed the following questionnaires: the Facebook Questionnaire, Australian Personality Inventory, Rosenberg’s self-esteem scale, 16-item Narcissistic Personality Inventory, the UCLA Loneliness Scale Version 3 | - Narcissism was not associated with time spent on Facebook per day or number of Facebook friends |
| Schwartz, 2010 (Schwartz M: The usage of Facebook as it relates to narcissism, self-esteem, and loneliness, unpublished) | - 218 undergraduate students (86 males, 127 female, 5 unspecified. Mean age = 21) completed questionnaires on Facebook usage, Rosenberg Self Esteem Scale, Narcissistic Personality Inventory-16, Three-Item Loneliness Scale | - Narcissism positively correlated with number of Facebook friends but not Facebook usage |
| Horton et al., 2014 [79] | - Experiment one: 88 male undergraduate students (mean age = 19.8) assigned randomly to one of three conditions: 1) agentic Facebook condition (engaging in their own Facebook profile), 2) communal Facebook condition (viewing what others have posted on Facebook), 3) control condition. Participants completed the Narcissistic Personality Inventory, 10-item Rosenberg Self-esteem scale, demographics, and questions on Facebook use  - Experiment two: 218 participants recruited from a college and university (141 men, 76 women, mean age = 19.6) engaged in agentic, communal, or control activities on and without a computer | - Narcissism positively correlated with self-reported daily Facebook activity in both experiments |
| Pettijohn et al., 2012 [112] | - 200 undergraduate students (65% female and 35% male, mean age = 19.9) completed the Facebook Intensity Scale, Friendship Contingent Self-Esteem Scale, and Narcissistic Personality Inventory (NPI-16), Ten-Item Personality Inventory | - Narcissism positively correlated with number of Facebook friends but not Facebook intensity |
| Weiser, 2015 [98] | - 1204 participants were recruited from Amazon MTurk (65% female, mean age = 32.8) and completed surveys on social media use and the 40-Item Narcissistic Personality Inventory (NPI-40) | - Narcissism predicts greater selfie posting frequency |
| Walters and Horton, 2015 [80] | - 80 male undergraduate students (age range = 18-22) were administered an online survey on Facebook usage, self-esteem, narcissism, and daily life experiences (filler items). | - Narcissism positively associated with Facebook use |
| Chen, 2014 [110] | -209 young adult Facebook users (71.3% female, 76.6% white, mean age = 19.9 years) completed surveys on their Facebook usage, Big Five Personality traits, and an adapted Narcissism Personality Inventory | - Narcissism positively associated with number of Facebook friends |
| Kojouri, 2015 (Kojouri C: Using Facebook to self-enhance: narcissism and psychological outcomes, unpublished thesis) | - A sample of 218 adolescents (109 female and 109 male, mean age = 14.6 years) completed an online survey on Facebook use, the 40- Item Narcissistic Personality Inventory, and psychological well-being | - Narcissism positively associated with how long adolescents had held a Facebook account, number of Facebook friends, the number of Facebook group membership, and number of groups that they were the administrator for |
| Weiss, 2013 [139] | - 171 college students aged 18-30 completed a demographic form on their social media usage, Leibowitz Social Scale – Self Report, the Rosenberg Self Esteem Scale, and the Narcissistic Personality Inventory | - Narcissism not associated to an individuals’ total time spent on their social media site or number of status updates |
| Ryan and Xenos, 2011 [95] | - 1324 adult Australian Internet users between the age of 18 and 44 years old (1158 Facebook users and 166 Facebook non-users) who completed the Big Five Inventory, Narcissistic Personality Inventory (NPI-29), Revised Cheek and Buss Shyness Scale, and Social and Emotional Loneliness Scale for Adults – Short Version. Facebook users completed Facebook usage questionnaire.  - Facebook users (460 men and 698 women); Facebook non-users (96 men and 70 women) – mean age group 25-34 for both | - Narcissism and exhibitionism positively associated with preferences for photo and status updates on Facebook  - Compared to Facebook non-users, Facebook users are more likely to be extraverted, narcissistic, and high in exhibitionism |
| Buffardi and Campbell, 2008 [117] | - 129 undergraduate Facebook page owners (100 females, mean age = 19.0) completed a demographic questionnaire, 40-item Narcissistic Personality Inventory (NPI, Raskin and Terry, 1988).  - The Facebook page owners provided their Facebook page to be rated by 128 undergraduate raters (86 females, mean age = 19.4) | - Higher narcissism correlated with greater Facebook interactions, greater self-promotional content on Facebook profiles, and attractiveness of the main photo |
| Arpaci et al. 2018 [99] | - Convenience sampling used  - 179 undergraduate students recruited from a Turkish university (50.28% female, mean age = 20.6) completed online surveys on Narcissistic Personality Inventory 16-Item, Selfie-posting scale, demographics and social media use | - Narcissism positively associated with selfie-posting in males but not females |
| Singh et al. 2018 [81] | - 124 social media users (mean age = 34.3, 75% male and 25% female) completed the 13-item Narcissistic personality inventory, social media usage, motivations for social media usage | - Narcissistic traits positively associated with greater social media usage, and greater tag/comment/like behaviors on social media sites.  - Narcissism correlated with greater selfie posting/sending frequency, self-rated selfie attractiveness, greater number of Facebook friends, Facebook updates, number of Twitter followers, frequency of tweets |
| Bibby, 2008 [96] | - 174 university students (mean age 20.5) completed questionnaires on social media use, Rosenberg’s Self Esteem Scale, an adapted Functional Social Support questionnaire, Narcissistic Personality Inventory, and the Big Five personality traits | - Higher level of exhibitionism-narcissism associated with greater usage of social networking sites for romantic purposes |
| Hawk et al. 2019 [119] | - Longitudinal study  - 160 girls and 147 boys (mean age = 12.9) completed the Childhood Narcissism Scale, and measures on social rejection, attention seeking, social media disclosure, problematic social media, and smartphone stress in two time intervals February 2015 and February 2016 | - Earlier adolescent narcissism predicted later social media disclosure, problematic use, and smartphone stress, via increased attention-seeking |
| Andreassen et al. 2017 [120] | - Cross sectional convenient sample of 25,532 Norwegians (8, 234 males and 15,298 females; mean age = 35.8 years old) completed the Bergen Social Media Addiction Scale, the Narcissistic Personality Inventory-16, and the Rosenberg Self Esteem Scale | - Narcissism was positively associated with addictive social media use |
| Biolcati and Passini, 2018 [100] | - 237 participants (20.7 male and 79.3% female, mean age = 24.0) filled out questionnaires on self-posting behaviors, self-posting motives, 16-item version of the Narcissistic Personality Inventory, the Rosenberg Self-Esteem Scale | - Narcissism positively correlated with selfie-posting and importance to likes received on social media |
| Kircaburun and Griffiths, 2018 [82] | - 772 university students (64% female, mean age = 20.7) completed the following questionnaires: Dark Triad Dirty Dozen Scale, Short Sadistic Impulse Scale, Spitefulness Scale, and an adapted version of the Bergen Facebook Addiction Scale. | - Higher narcissism is associated with greater social media usage |
| McKinney et al. 2012 [113] | - 233 undergraduate students (62% women, 38% men, mean age = 19.7) completed questionnaires on openness toward self-disclosure (ex: “I enjoy letting people know things about me”), social media usage, and the Narcissistic Personality Inventory | - Higher narcissism associated with greater self-reported number of Facebook friends |
| Carpenter, 2012 [124] | - 294 participants (mean age = 23.3, 68% female) completed questionnaires on demographics, Facebook use, Grandiose Exhibitionism and Entitlement/Expoitiveness subscales of the Narcissistic Personality Inventory, and the Rosenberg self-esteem scale | - Scores on Grandiose Exhibitionism-Narcissism predicted self-promoting Facebook behaviors, Facebook friends count, frequency of accepting strangers as Facebook friends |
| Ong et al., 2011 [115] | - 275 adolescents with a Facebook account (165 females, 109 males, one did not report gender, mean age = 14.2) completed the Narcissism Personality Questionnaire for Children-Revised, Extraversion subscale of the NEO Five-Factor Inventory, and Facebook usage | - After controlling for extraversion, narcissism predicted higher self-ratings of their Facebook profile picture as being more attractive and higher frequency of Facebook status updates |
| Wang et al., 2012 [116] | - 265 undergraduate students (mean age = 20.2) completed questionnaires on social media use, Big Five Personality Inventory, Rosenberg Self-Esteem, Narcissism Personality Inventory, Sensation Seeking Scale | - Narcissism associated with greater likelihood of upload attractive self-photos on social media and more frequent status updates |
| McCain and Campbell. 2018 [83] | - Data from 62 research samples (N = 13,430) are meta-analyzed with respect to the relationships between grandiose and vulnerable narcissism and (a) time spent on social media, (b) frequency of status updates/tweets, (c) number of friends/followers, and (d) frequency of posting selfies on social media | - Grandiose narcissism positively related to time spent on social media, frequency of status updates, number of friends/followers, and frequency of selfies posted on social media |
| Frederick and Zhang, 2019 [140] | - 397 Amazon MTurk participants (236 female, 158 male, 3 unknown; mean age = 29.4) completed a demographic survey, a social media usage survey, and the Narcissistic Personality Inventory | - Narcissism was not associated with greater social media usage |
| Alloway et al. 2014  [125] | - 410 participants (25 % male, age range 18 – 50 years) completed questionnaires on Facebook usage, Interpersonal Reactivity Index, and Narcissistic Personality Inventory–16 | - Higher narcissism scores associated with greater frequency of posting, tagging, and commenting on photos |
| Zell and Moeller., 2017 [134] | - 311 participants (77.4% female, mean age = 26.5) completed an online survey on their Facebook activity and the Narcissistic Personality Inventory -13 | - Entitlement/Expoitiveness-narcissism correlated with greater importance placed on getting a response on Facebook, looking popular on Facebook, and becoming angry and desire at retaliating against people who did not respond to one’s status updates  - Narcissistic Grandiose Exhibitionism correlated with reporting having cared about responses and tried to look popular |
| McCain et al., 2016 [101] | - In Study 1, 348 adults (mean age: 31.85, 49% female) recruited from Amazon MTurk completed the Narcissistic Personality Inventory-13, the Hypersensitive Narcissism Scale, the Short Dark Triad questionnaire, the Rosenberg Self-Esteem Scale, demographics and several questions about selfie behaviour online, and Instagram use  - Study 2, 491 undergraduate students (mean age: 18.8, 78.6% female) completed the same set of questionnaires as described above; a subset of Study 2 participants also provided the researcher a selfie and access to their Instagram and Iconosquare pages, which were rated for narcissism | - Narcissism is associated with taking and posting more selfies, experiencing more positive affect when taking selfies, and greater motivation for self-presentation motives in both studies |
| Fox and Rooney, 2015 [84] | - 800 men (mean age = 29.3) completed the following surveys: Self-Objectification Questionnaire, dark triad traits (narcissism, psychopathy, Machiavellianism), social media usage, posting/editing photo behaviors | - Narcissism associated with spending more time on social media  - Narcissism predicted number of selfies posted, as well as greater photo-editing behaviors in self-photos |
| Marshall et al., 2020 [133] | - Study 1: 614 participants (57% female, mean age = 30.6) completed the following questionnaires: Big Five Personality Traits, 13-item version of the Narcissistic Personality Inventory, social media usage, motives for using Twitter  - Study 2: 503 participants (59% female, mean age = 37.32) completed the Dark Triad questionnaire, social media usage and Berkley Personality Profile | - Narcissistic traits positively associated with greater motivation of using Twitter for self-promotional purposes (ex: career promotion, attention seeking, social connections) |
| Charoensukmongkol, 2016 [109] | - 300 university students (39.7% male, 60.3% female) completed questionnaires on selfie-like behaviors (ex: “taking selfies make me happy”), narcissism, attention-seeking, self-centered behavior, loneliness, social media usage, demographics, self-rated friendliness (“I consider myself a sociable person”), and peer pressure (“peers in my group like to show off about being better than others in the group”) | - Narcissism positively associated with selfie-liking behaviors |
| Giordano et al., 2019  [102] | - 627 undergraduate students (283 males and 344 females, mean age = 22.8) completed a cross-sectional survey on demographics, 40-item Narcissistic Personality Inventory, Smartphone Addiction Inventory-Italian version, selfie-related behaviors (i.e. number of selfies taken in one day, number of selfies posted in one day) | - Greater narcissism associated with increased selfie-related behavior (number of selfies taken and number of selfies posted in one day) |
| Lyvers et al., (2022) [121] | - 217 adults (mean age 22.33 years, 167 females) completed questionnaires on demographics, Narcissistic Personality Inventory 16, Toronto Alexithymia Scale 20, Social Interaction Anxiety Scale, Social Media Disorder Scale, Internet Addiction Test | - Narcissism and social anxiety were significant predicators for both social media and internet addiction symptoms |
| Boursier et al., (2020) [143] | - 570 young adults (66.8% female, mean age = 24.4 years) completed the Pathological Narcissism Inventory, Objectified Body Consciousness Scale, Selfie-expectancies Scale, and a measure of selfie-engagement | - Pathological narcissism not a significant predicator for selfie-engagement  - Selfie-engagement was predicted by body surveillance and positive selfie-expectancies (i.e. self-confidence, self-presentation, sexual desire about selfie-taking) in both men and women |
| Brailovskaia et al., 2020b [129] | - 449 Facebook users (72.2% women, mean age = 31.07 years) completed the brief German Narcissistic Personality Inventory, Facebook usage, Facebook Intensity Scale, modified Facebook flow questionnaire (e.g., "Time flies when I am using the Facebook"), brief version of the Bergen Facebook Addiction Scale | - Narcissism positively correlated with Facebook addiction |
| Casale and Fioravanti, 2018 [130] | - 535 undergraduates (50.08% female, mean age 22.70) completed the Italian adaptation of the Narcissistic Personality Inventory-16, Hypersensitive Narcissism Scale, Admiration-seeking Behavioural Scale, a Need to Belong Scale, and the Bergen Facebook Addiction Scale | Grandiose narcissism was positively associated with Facebook addiction levels, which are mediated by need for admiration and need to belong  Convert narcissism was not significantly associated with Facebook addiction levels |
| Ljepava et al., 2013 [85] | - 106 undergraduates (91 females, mean age: 21.34) filled out the Sharabany Intimate Friendship Scale, Facebook Peer Usage Questionnaire, General Trust subscale of the Couch's Trust Inventory, Self Disclosure Scale, Narcissism Personality Inventory, and Hypersensitive Narcissism Scale | - Frequent Facebook users scored higher on overt narcissism than Facebook non-users; in contrast, Facebook non-users had lower tendency to self-close and had higher covert narcissistic traits |
| Winter et al., 2014 [118] | - 172 adults (102 female, mean age = 25.95) filled out the NEO Five Factory Inventory scale, Narcissistic Personality Inventory-16, Mielke's self-efficacy scale, questions that assess need to belong and motivation for affiliating with others, questions that assess need for popularity, and Facebook usage (i.e., number of Facebook friends, number of status updates posted in the last four weeks) | - Narcissism is positively related to number of posted status updates, deeper self-disclosure of such updates, and more self-promotional content in status updates |
| Shane-Simpson et al., 2020 [103] | - 730 undergraduate students (mean age = 19.61, 61% female) completed the Narcissistic Personality Inventory-13, Hypersensitive Narcissism Scale, selfie posting behaviours, and demographics questionnaire | - Selfie posting frequency was predicted by grandiose narcissism |
| Brailovskaia and Bierhoff., 2016 [88] | - 72 Russian (37 female, mean age = 25.18) and 122 German Facebook users (70 female, mean age = 23.33) completed the Narcissistic Personality Inventory, Narcissistic Inventory-Revised, Facebook usage. Participants also had their Facebook profiles analyzed. | - In both the Russian and German samples, narcissism was positively correlated with being a part of more Facebook groups, more links shared, more Facebook friends, more status updates, and more messages written and received |
| Sorokowski et al., 2015 [104] | - In study 1, 748 people (355 women, mean age: 21.64) completed the Polish adaptation of the Narcissistic Personality Inventory and self-reported their selfie sharing activities during the previous month.  - In study 2, 548 Facebook users (330 women, mean age: 23.72) completed the same questionnaire and had their photos posted on Facebook page analyzed by research assistants. | - Overall narcissism scores positively predicted posting own selfies, selfies with a partner, and group selfies in men but not women |
| Halpern et al., 2016 [105] | - 314 adults (48.7% male, 51.3% female) completed the Narcissistic Personality Inventory, self-reported levels of extroversion and openness to experience, and frequency of selfie activities across two timepoints, with a year in between. Participants also reported age and gender. | - Frequency of selfie-taking positively correlated with narcissism over time. |
| Balcerowska et al., 2019 [132] | - 486 participants (64.4% women, mean age = 21.56) completed the Bergen Facebook Addiction Scale, the Narcissistic Personality Inventory, and the Polish version of the Ten Item Personality Inventory | - A particular dimension of narcissism (passivity and dependency on others) may be a risk factor for Facebook addiction |
| Moon et al., 2016 [89] | - 212 (110 females, mean age = 28.8) active Instagram users completed the 13-item Narcissistic Personality Inventory, Instagram usage, selfie activities, frequency of profile picture updates and self-report of physical appearance ratings of their current profile picture | - Individuals higher in narcissism tend to post a greater proportion of selfies and self-presented photos (i.e. a photograph that one has taken with others, update profile pictures more frequently, rate their own profile picture as more physically attractive, and spend more time on Instagram compared to individuals lower in narcissism.  - In particular, Grandiose-Exhibitionism positively predicted frequency of selfie postings, profile picture updates, and more positive evaluation of one's own profile picture |
| Brailovskaia et al., 2020a [131] | - 327 adult Facebook users (72.8% female, mean age = 23.57) completed the abridged version of the Narcissistic Inventory (NR-R-36), Narcissistic Personality Inventory-13, the anxiety subscale of the Depression Anxiety Stress Scales 21, and the brief version of the Bergen Facebook Addiction Scale | - Both vulnerable and grandiose narcissism are positively correlated with Facebook addiction scores |
| Errasti et al., 2017 [90] | - 503 adolescents (231 females) completed the Basic Empathy Scale, Narcissistic Personality Inventory, Rosenberg Self-Esteem Scale, and emotional and empathic usage of Facebook and Twitter | - Narcissism-Exhibitionism is positively correlated with the frequency of Twitter usage, number of Facebook friends, and greater expression of emotions on Twitter and Facebook |
| Martingano et al., 2022 [91] | - 1253 adults (mean age = 27.6, 69.7% female) completed questionnaires on social media use, the 16-item Narcissistic Personality Inventory, 20-Item Toronto Alexithymia Scale, Interpersonal Reactivity Index, Facial Action Coding System- verified University of California set of Emotions Expressions | - Social media use is negatively correlated with self-report and performance measures of empathy and positively correlated with narcissism and alexithymia |
| Brailovskaia and Margraf, 2016 [92] | - 790 adult (mean age = 23.42, 562 women) Facebook users and 155 adult Facebook non-users (99 women, mean age = 25.28) completed the Subjective Happiness Scale, Satisfaction with Life Scale, German Resilience Scale 11, German Questionnaire Social Support questionnaire, Narcissistic Personality Inventory 13, Big Five Inventory 10,  Single-Item Self-Esteem Scale, Depression Anxiety Stress Scales 21, and social media usage | - Facebook users scored significantly higher on narcissism than non-Facebook users |
| Barry et al., 2019 [141] | - 100 undergraduate students (80 females, mean age = 19.93) provided their Instagram accounts to be "followed" by the researcher for 30 days. Participants also filled out a demographics questionnaire, the Pathological Narcissism Inventory, Narcissistic Personality Inventory, the Rosenberg Self-Esteem Scale, Physical Appearance Comparison Scale, and Sociocultural Attitudes Appearance Scale-30, and Fear of Missing Out Survey | - Narcissism was not significantly correlated with number of Instagram followers  - Grandiose narcissism was positively correlated with a greater proportion of Event selfies taken  - Nonpathological narcissism negatively correlated with proportion of Event selfies and collage "posies" taken (i.e. non selfie photos)  - Vulnerable narcissism positively correlated with greater proportion of affiliation "posies" taken |
| Koterba et al., 2021 [106] | - A total of 276 college students (218 females) (mean age = 20.03) completed the Narcissistic Personality Inventory-13 and reported the number of selfies that they have taken in the past week that was only of themselves and also those that included others | - Grandiose and exhibitionistic dimensions of narcissism predicted number of selfies taken alone for both males and females  - The top motive for taking selfies were for narcissistic reasons ("I think I am attractive and have no problem sharing that") versus other reasons (i.e. sharing and connecting, functional use, self esteem boosting, memory, conformity) |
| Brailovskaia and Margraf, 2019 [93] | - 328 Facebook users (66.2% women, mean age: 24.83) completed the unidimensional Subjective Happiness Scale, Depression Anxiety Stress Scale-21, Narcissistic Personality Inventory-40, Facebook usage, and had a copy of their Facebook account page saved on a computer in an university laboratory | - Narcissism is positively correlated with active Facebook use (e.g. writing and commenting on status updates, number of likes, Facebook friends, and number of uploaded photos and albums) |
| Reed et al., 2018 [122] | - 74 undergraduate students (55 female, mean age: 23.09 years) completed the Narcissistic Personality Inventory (NPI-40), Problematic Internet Use Questionnaire, demographics, and internet usage | - Initial levels of Problematic Internet Use predicted subsequent levels of narcissism four months later, but only for those who used primarily visually-based forms of social media (e.g., Instagram) vs verbally-based social media (e.g., Twitter) |
| Eşkisu et al., 2017 [144] | - 492 undergraduate students (356 women, mean age: 20.28 years) completed the Intended Purpose of Social Network Sites Scale, questions about Facebook usage, Rosenberg Self-Esteem Scale, Narcissistic Personality Inventory, and Big Five Inventory | - Narcissism scores did not differ between those who did or did not have a Facebook account or frequency of Facebook checking, frequency of status updates  - Narcissism scores differed significantly in terms of time spent on Facebook and number of Facebook friends. Narcissism scores were significantly higher for participants who spent more than 3 hours a day on Facebook vs participants who spent less than an hour. Narcissism scores were also higher for participants who had more than 300 Facebook friends vs those who had 151-300 friends. |
| Scott et al., 2018 [145] | - 264 participants (189 females, mean age = 31.65) completed on a questionnaire on Facebook activity, Narcissistic Personality Inventory Version-16, the Revised Cheek and Buss Shyness Scale, UCLA Loneliness Scale version 3, Liebowitz Social Anxiety Scale | - Narcissism was a significant predictor of the frequency of posting of photos in the pets, travel, sport, food, achievement, and the other category but not selfies  Narcissism positively predicted likelihood of posting photos of the category Pets |
| Sung et al., 2016 [107] | - 319 participants who identify as selfie-takers/posters completed the 13-item Narcissistic Personality Inventory, a question on selfie-posting frequency, and questions on selfie-posting intention | - Narcissism was correlated with three of the four selfie-posting motivations: attention seeking, communication, and entertainment, intention to post selfies in the future, and selfie-posting frequency |
| Burnell et al., 2020 [94] | Participants consist of two groups. Both groups completed questionnaires on narcissism and patterns of social media activity/usage. Study 1 is a community sample of 134 young adults (mean age: 22.15, 55% female). Study 2 consist of 814 undergraduate students (mean age = 20.76, 76% female). | - Grandiose narcissism was positively associated with active social media usage  - Entitlement/self-antagonism was associated with active use of social media usage in study 2 but not in study 1 |
| Lee et al., 2014 [114] | - 236 college students (54.2% female, 45.8% male, mean age = 20.6 years) completed the mini-IPIP scales to measure the Big Five Personality traits, Narcissistic Admiration and Rivalry Questionnaire, Facebook usage, Facebook activity, and demographics variables | - Narcissism was positively associated with frequency of status updates on Facebook wall |
| Błachnio et al., 2016 [128] | - 653 adults (73% female, mean age = 21.63 years) with a Facebook profile completed the Facebook Motivation and Importance Scale, Facebook Intensity Scale, Narcissistic Personality Inventory, Rosenberg's Self Esteem Scale | - Narcissism positively predicted Facebook personal importance (e.g., "I want to express and present myself"), instrumental Facebook use (e.g., "I use Facebook for study-related purposes"), and Facebook intensity (i.e. the extent in which Facebook is used and integrated into daily activities and emotional involvement in Facebook) |
| Martin, 2020 [146] | - 236 undergraduate students (164 women, mean age - 19.81) completed the Revised Competitiveness Index, Attitude Toward Competition Questionnaire, and the Narcissistic Personality Inventory. Participants also had a screenshot of their Facebook profile & last ten pictures posted analyzed. | - Men and women scoring higher on vanity posted more athletic photos on Facebook  - Both men and women scoring higher in authority/entitlement posted more sexual photos  - Individuals scoring lower in vanity posted more attractive photos, but only in men |
| Liu and Baumeister, 2016 [268] | - Meta-analysis of 80 studies yielding 143 effect sizes on the effects of self-esteem, narcissism, and loneliness on social media usage | - Total social media usage is higher in individuals high in narcissism  - High narcissism linked to higher social media usage such as status updates, posting photographs, interacting/commenting on others updates, and total online friends. |
| Gnambs and Appel, 2018 [86] | - A three-level, random-effects meta-analysis including 289 effect sizes from 57 studies (total N - 25 631) on the association between narcissism and social networking behaviour | - A small to moderate effect was found for grandiose narcissism and increased social media usage that replicated across different social networking platforms, respondent characteristics, and time |
| Wang, 2019 [108] | - 722 undergraduate students (419 females, mean age = 22.36) completed the 16-item version of the Narcissistic Personality Inventory, 12-item subscale of the NEO Five-Factor Inventory, 23-item Body-Esteem Scale for Adolescents and Adults, and questions on selfie-editing and self-posting frequencies | - Narcissism is positively related to selfie-editing frequency |
| Casale et al., 2016 [123] | - 535 undergraduate students (50.9% female, mean age: 22.73) completed the 16-item Narcissistic Personality Inventory, the Hypersensitive Narcissism Scale, and the Generalized Problematic Internet Use Scale-2 | - Vulnerable narcissists reported higher levels of problematic social media usage than grandiose narcissists and non-narcissists  - No significant differences for problematic social media usage between grandiose narcissists and non-narcissists |
| Lee and Sung, 2016 [135] | - 314 social media users who has posted selfies on social media (mean age: 29 years, 221 female) completed the 13-Item Narcissistic Personality Inventory, questions on how they are involved in the feedback on their selfies, questions on how participant observe other peoples' seflies, and questions on particpants' attitude toward selfie-posting behaviour, and questions on intention to post selfies | - Participants with higher narcissistic scores are more involved in the feedback (e.g., comments and likes) received on their selfies and more observant of others' selfies  - Participants higher in narcissism regard selfie-posting more positively and are more motivated in posting more selfies in the future  - Narcissism is not associated with greater likelihood of posting a comment or "likes" on other peoples' selfies |
| Pantic et al., 2017 [127] | - 244 undergraduate students (157 female, mean age: 21.98 years) completed a demographics questionnaire, Internet Addiction Test, Rosenberg Self-Esteem Scale, the 16-item Narcissistic Personality Inventory | - Narcissism is positively correlated with internet addiction scores and number of Facebook selfies  - Self-esteem is negatively correlated with internet addiction scores |
| Brailovskaia et al., 2019 [126] | - 112 Facebook users (71.4% women, mean age = 49.43) who were also inpatients at a psychosomatic rehab clinic completed questions on their general daily Internet use, Facebook usage, Bergen Facebook Addiction Scale, and Narcissistic Personality Inventory | - Facebook Addiction Disorder is positively associated with duration of Facebook use and narcissistic traits |
| Barry et al., (2017) [142] | - 128 undergraduate students who had an active Instagram account (19 males, 109 females, mean age = 20.46) completed the Pathological Narcissism Inventory, Narcissistic Personality Inventory, Rosenberg Self-Esteem Scale and had their Instagram activities and pictures evaluated by independent coders | - Narcissism is not significantly associated with overall number of selfies posted  - Vulnerable narcissism is positively associated with a higher proportion of posts that were physical appearance selfies, grandiose narcissism negatively associated with proportion of posts that were affiliation selfies (i.e. selfies with others); nonpathological narcissism is positively correlated with the proportion of selfie collages |
