# Supplementary material for: I tweet, therefore I am: a systematic review on social media use and disorders of the social brain
Source: BMC Psychiatry. 2025 Feb 3;25:95. doi: 10.1186/s12888-025-06528-6 (PMC11792667; doi:10.1186/s12888-025-06528-6)
Supplement: Supplementary file 4 — Supplementary Material 4. [file 12888_2025_6528_MOESM4_ESM.docx]

**Supplementary Table 4.** Relationships of social media usage with body image perturbations (aside from BDD) and eating disorders

| References | Methods | | Findings |
| --- | --- | --- | --- |
| Tiggemann and Slater, 2017 [193] | - 438 girls (mean age = 13.6 years) completed questionnaires at two time periods when they were in the first two years of high school  - participants completed questionnaires on Facebook use and Body Image Concerns | | Number of Facebook friends, but not Facebook usage, prospectively predicted greater drive for thinness in a sample of adolescent girls |
| Butkowski et al., 2019 [189] | - 177 female young adult (age 18-30) Instagram users who has posted selfies were recruited from MTurk  - participants completed questionnaires on: 1) selfie feedback investment (i.e. degree to which participants value feedback on their posted Instagram selfies), body image disturbance, body dissatisfaction, drive for thinness, bulimic tendencies, body surveillance (ex: “during the day, I think about how I look many times”), Instagram usage, and demographics | | Instagram use frequency not associated with greater body dissatisfaction or drive for thinness |
| Cohen et al., 2017 [192] | - 259 women (mean age = 22.97) completed questionnaires on demographics, social media usage, and the Internalisation-General subscale of the Sociocultural Attitudes Towards Appearance Questionnaire–Version 3, Physical Appearance Comparison Scale, The Appearance Evaluation subscale of the Multidimensional Body-Self Relations Questionnaire-Appearance Scales, The Body Surveillance Subscale of the Objectified Body Consciousness Scale, and the Drive for Thinness Subscale of the Eating Disorder Inventory-3 | | Instagram users scored higher on the body surveillance (ex: “during the day, I think about how I look many times”) scale than non Instagram users  Drive for thinness was not associated with time spent on social media but following “health and fitness” Instagram accounts  Greater Facebook appearance exposure (ex: updating one’s profile photo and viewing friends’ photos) positively associated with greater body surveillance |
| Hendrickse et al., 2017 [164] | - 185 female college students (mean age = 21.04) with an Instagram account completed questionnaires on Instagram photo activity, appear-related comparison on Instagram, Intrasexual Competition Scale, and drive for thinness via subscale from Eating Disorder Inventory | | Instagram photo-based activities positively predicted both drive for thinness and body dissatisfaction through the mediating variable of appearance-related comparisons. |
| Stronge et al., 2015 [165] | - 11 017 adults (mean age = 49.23; 6,883 women, 4,134 men) completed questionnaires on demographics, body satisfaction, and Facebook use | | Facebook users reported significantly lower body satisfaction than non-users. |
| Howard et al., 2017 [166] | - 922 female participants (mean age = 21 years old) completed the Body Shape Questionnaire, Eating Disorder Examination Questionnaire, social media usage, and Social Media Reassurance seeking Scale | | More frequent Facebook usage associated with greater body dissatisfaction |
| Meier and Gray, 2014 [167] | - 103 adolescent female students (mean age = 15.4) completed the following questionnaires: demographics and body mass index, Sociocultural Internalization of Appearance Questionnaire for Adolescents, the Physical Comparison Scale (PACS), Weight Satisfaction subcale for Adolescents and Adults, Drive for Thinness subscale on the Eating Disorder Inventor, Self-Objectification Questionnaire, total Internet and Facebook usage, Facebook Appearance-related Exposure | | Frequency of Facebook appearance exposure (i.e. Facebook-photo related activities) activities positively correlated with internalization of the thin ideal, self-objectification, and drive for thinness, and negatively correlated with weight satisfaction |
| Murray et al., 2016 [168] | - 383 (70.2 percent female) undergraduate students (mean age = 23.1 years) who completed self-report questionnaires on social media usage, the Dutch Eating Behavior Questionnaire, and Body Esteem Scale for Adolescents and Adults | | Greater use of social media was associated with more weight and appearance dissatisfaction, which was in turn associated with more severe disordered eating. |
| Santarossa and Woodruff, 2017 [176] | 147 young adults (55% female, 45% male, age range 18-27) completed a series of online surveys which measured social media usage, problematic social media usage, body image, self-esteem, and eating disorder symptoms | | Time spent on social networking sites was positively associated with greater eating disorder symptoms |
| Tiggemann and Slater, 2014 [174] | - 189 girls (mean age = 11.5) completed questionnaires on magazine/television exposure, internet exposure, body image concerns, and dieting behaviors and status | | Time spent on these social networking sites positively correlated with internalization of thin idea, body surveillance, and dieting behavior. |
| Walker et al., 2015 [212] | - 128 college-aged women completed questionnaires on disordered eating, Facebook Intensity Scale (i.e. emotional connection to Facebook and integration of Facebook into one’s daily life), Online Physical Appearance Comparison Scale, Online Fat Talk Scale, body mass index, Beck Depression Inventory II, State-Trait Anxiety Inventory, Multidimensional Perfectionism Scale, (Negative) Urgency, (Lack of) Premeditation, (Lack of) Perseverance, and Sensation Seeking (UPPS-P) Impulsive Behavior Scale: Negative Urgency, General Self-Efficacy Scale | | Facebook intensity associated with increased online physical appearance comparison, which in turn was associated with greater disordered eating |
| Yellowlees et al., 2019 [175] | A total of 152 females (mean age = 22.4 years) with ED symptoms of clinical severity completed self-report questionnaires on: social media use, selfie-behavior, Eating Disorder Examination Questionnaire, body mass index, Body Dissatisfaction subscale of the Eating Disorder Inventory, Body Checking Questionnaire, Body Image Avoidance Questionnaire, the Rosenberg’s Self Esteem Scale | | Higher levels of social media usage positively correlated with eating disorder symptoms  Greater offline selfie-taking correlated with greater eating disorder symptom severity via greater body checking behaviors |
| Tiggemann and Anderberg, 2020 [185] | - 305 women (mean age = 25.3 years) recruited from TurkPrime were randomly assigned to viewing one of three sets of Instagram images: “Instagram vs reality” images (curated and realistic images were paired side-by-side”, “idealized” image side only, or realistic images side only. Participants also completed questionnaires on social networking usage, body dissatisfaction, body appreciation (ex: “despite my flaws, I accept my body for what it is”), 3-item State Appearance Comparison Scale of Tiggemann and McGill (2004) | | Viewing “idealized” and curated Instagram images was associated with increases in body dissatisfaction, but not the “Instagram vs reality” images (where the curated and realistic images were put side by side) or the realistic images |
| Turner and Lefevre, 2017 [177] | - 680 women (mean age = 24.7) completed online surveys on social media use, dietary choices, orthorexia nervosa questionnaire (ORTO-15), and demographics | | Higher Twitter and Instagram use were associated with increased orthorexia nervosa symptoms |
| Sidani et al., 2016 [179] | - 1765 participants completed questionnaires on eating concerns (i.e. eating disorder symptoms), social media usage, and demographics | | Both volume (i.e. time per day) and frequency of social media use (i.e. visits per week) were positively associated with self-reports of eating concerns (ex: preoccupation with food, dysfunctional eating patterns, preoccupation with one’s weight) in a sample of young adults |
| Mabe et al., 2014 [178] | - study one: 1960 college-aged (mean age = 18.4) women completed self-report questionnaires on Facebook use and disordered eating (Eating Attitude Test-26)  - study two: 84 women, from study 1, who identified as weekly Facebook users, were randomly assigned to use Facebook for an alternate site for 20 minutes. Participants also completed surveys on demographics, preoccupation with weight/scale/shape, and State Trait Anxiety Inventory State scale | Facebook use positively associated with greater disordered eating  Participants with greater eating disorder symptoms placed greater importance of receiving “likes” and comments on status, and more likely to compare their own photos to female friends’ photos | |
| Tiggemann and Slater, 2013 [169] | - 1087 adolescent girls (aged 13-15) completed questionnaires on measures of Internet exposure and body image concerns (i.e., Sociocultural Attitudes Toward Appearance Questionnaire and Objectified Body Consciousness Scale - Youth) | - compared to Facebook non-users, Facebook users scored higher on the Internalization of Thin ideal, Body Surveillance, and Drive for Thinness  - among the Facebook users, number of Facebook friends was significantly correlated with internalization of thinness ideal, body surveillance, and drives for thinness scores | |
| Hummel and Smith (2015) [188] | - 185 students (78% female, mean age: 18.73 years) participated in a two-part study.  Participants were asked to fill out questionnaires on two separate occasions separated by about four weeks. Additionally, participants' Facebook status updates and comments on such updates were recorded. Participants completed the following questionnaires: Eating Disorder Examination Questionnaire (EDEQ-4) and one item from the Maladaptive Facebook Questionnaire to measure Facebook feedback seeking | - participants who received extremely negative comments in response to personally revealing status updates (i.e., status updates about one's personal life) were more likely to report disordered eating concerns four weeks later | |
| McLean et al., (2015) [186] | - 101 grade seven girls (mean age = 13.1 years) completed questionnaires on demographics, digital media and social media use, photo activities (i.e., taking and sharing images online), frequency of selfie-taking, frequency of selfie-sharing, photo investment (i.e., efforts taken in choosing photos of themselves to share on social media and concern about such pots), photo manipulation, Body Dissatisfaction subscale on the Eating Disorders Inventory, two items from the Eating Disorder Examination Questionnaire to assess overvaluation of shape and weight (i.e., "Over the past 28 days, has your body shape influenced how you think about (judge) yourself as a person?", Dutch Eating Behaviour Questionnaire Restraint subscale, Sociocultural Attitudes Towards Appearance Questionnaire-4 | - Girls who regularly share selfies on social media, relative to those who did not, reported significant highly overvaluation of shape and weight, body dissatisfaction, dietary restraint, and internalization of the thin idea  - among girls who shared selfie on social media, higher selfie- investment and selfie-manipulation were associated with greater body-related and eating concerns | |
| Holland and Tiggemann (2017) [187] | - 101 women who regularly post fitspiration images (mean age: 26.05) and 102 women (mean age = 30.51) who regularly post travel images on Instagram completed the Drive for Thinness, Bulimia, and Body Dissatisfaction subscales of the EDI (Eating Disorder Inventory) and the four-item Emotional Element subscale of the Obligatory Exercise Questionnaire. | - women who post fitspiration images scored significantly higher on drive for thinness, bulimia, drive for muscularity and compulsive exercise.  - 17.4% of the fitsinspiration group were at risk for diagnosis of eating disorder, compared to 4.3% of the travel image group | |
| Wilksch et al., (2020) [171] | - 996 adolescents (534 girls; mean age = 13.08) completed the Eating Disorder Examination-Questionnaire (EDE-Q) (questionnaire that measures disordered eating cognition), social media usage, and Project Eating Among Teens questionnaire (questionnaire that measures disordered eating behaviours) | - a higher number of social media accounts was associated with higher disordered eating scores for both cognition (e.g., concern about weight/shape/ eating) and behaviours (i.e. skipping meals, binge eating)  - greater daily time spent using Instagram was associated with higher Global EDE-Q scores and disordered eating behaviours in girls  - spending more time on Snapchat was associated with increased likelihood of reporting disordered eating behaviours in girls but not boys | |
| Rodgers et al., (2020) [170] | - 681 adolescents (49% female, mean age = 12.76 years) completed questionnaires on social media usage, self-esteem, Center for Epidemiological Studies Depression Scale-revised version for adolescents (CESDR-10), the Muscular/Athletic Internalization subscale of the Sociocultural Attitudes Toward Appearance Questionnaire-4, Internalization General subscale of the Sociocultural Attitudes Toward Appearance Questionnaire-3, a modified version of the Upward Physical Appearance Comparison Scale, weight and shape concern subscales from the Eating Disorders Examination questionnaire, restrained eating subscale of the Dutch Eating Behaviours Questionnaire, Body Change Inventory, and BMI measures | - social media usage is correlated with lower self-esteem, higher depressive symptoms, higher internalization of appearance ideals, higher tendency to engage in appearance comparison, greater dietary restraint, increased body dissatisfaction and changes in muscle-building behaviours in girls and boys | |
| Yao et al., (2021) [184] | - 838 college women (mean age = 19.97) completed questionnaires on body image comparisons on social networking sites, body shame subscale of the Chinese version of the Objectified Body Consciousness Scale, the Body Appreciation Scale-2, body mass index, Restrained Eating subscale of the Chinese version of the Dutch Eating Behaviours Questionnaire | Body image comparison on social networking sites is positively associated with restrained eating; this association is mediated by body shame. | |
| Fardouly and Vartania., 2015 [172] | - 227 female undergraduates (mean age: 19.13 years) completed questions on Facebook usage, Facebook appearance comparisons in general (three questions adapted from the Physical Appearance Comparison Scale), frequency and direction of appearance comparison of female target groups on Facebook (e.g., "When looking at photos of the following people on Facebook, how often do you compare your body to theirs?", two subscales of the Eating Disorder Inventory (Body Dissatisfaction subscale, Drive for Thinness subscale) | Frequency of Facebook usage was positively associated with body dissatisfaction and drive for thinness, which is mediated by appearance comparison in general, appearance of comparison to friends and distant peers, and upward comparison to distant peers and celebrities | |
| Kim and Chock, 2015 [191] | - 186 individuals (119 female, mean age = 19.75) completed a revised version of Utz and Beukeboom's SNS use for grooming scale, self-report number of Facebook friends, time spent on Facebook per day, Physical Appearance Comparison Scale, Drive for Thinness subscale of the Eating Disorder Inventory, Drive for Muscularity Scale, and demographics | - viewing and commenting on peers' profiles, and not time spent on social media, were associated with body image concerns (i.e., greater drive for thinness) for both females and males | |
| Cohen et al., 2018 [194] | - 259 women (mean age = 22.97) completed questionnaires on demographics, social networking site use, the Photo Activities measure, the Internalization-General subscale of the Sociocultural Attitudes Toward Appearance Questionnaire-Version 3, the Appearance Evaluation subscale of the Multidimensional Body-Self Relations Questionnaire-Appearance Scale, the Body Surveillance subscale of the Objectified selfie-objectification, the Drive for Thinness and Bulimia subscale of the Eating Disorder Inventory-3 | - greater selfie investment, but not general social media usage, is associated with increased body dissatisfaction and bulimia symptoms | |
| Jiotsa et al., 2021 [183] | - 1331 participants (1300 female, mean age: 24.2, 1138 non-clinical participants and 193 patients with eating disorders) completed questionnaires on social media usage, and the Body Dissatisfaction subscale and Drive for Thinness subscale from the Eating Disorder Inventory Scale, and the Sick-Control-One-Stone-Fat-Food self-questionnaire for screening eating disorders | - frequency of comparing one's physical appearance to people followed on social media are increased with greater body dissatisfaction and drive for thinness scores | |
| Smith et al., 2013 [180] | - 232 female adults (mean age: 18.72) completed the Maladaptive Facebook usage Questionnaire, Eating Disorder Inventory, Eating Disorder Examination Questionnaire-4, and the Depressive Interpersonal Relationship Inventory—Reassurance Seeking Subscale over on two separate occasions, separated by a period of two to four weeks | - maladaptive Facebook usage predicted increases in bulimic symptoms and over-eating episodes four week later. | |
| Fardouly et al., 2018 [215] | - 276 young women ( mean age: 22.83 years) completed questions on Instagram usage, frequency of viewing fitspiration images in Instagram, appearance comparison, the behaviours on Instagram, the Internalization-General subscale of the Sociocultural Attitudes Toward Appearance Questionnaire-3, the Upward and Downward Appearnce Compairson Scale, the Body Dissatisfaction suscale and Drive for Thinness subscale on the Eating Disorder Inventory, the Self Objectification Questionnaire | - greater Instagram use is associated with greater self-objectification  - more frequently viewing fitspiration images on Instagram s associated with greater body image concerns | |
| Kaewpradub et al., 2017 [190] | - 620 high school students (mean age: 15.7 years, 60.3% female) completed questionnaires on internet and media usage, the Body-Esteem Scale for Adolescents and Adults, Drive for Muscularity Scale, Rosenberg self-esteem scale, Eating Attitudes and Behaviors questionnaire (EAT-26), and eating behaviours at risk of obesity questionnaire. Participants also had their nutrition status assessed. | - use of internet and social media for material related to body image was not significantly related to eating and body image problems after adjusting for age, gender, and general use of internet and social media | |
| Mingoia et al., 2017 [173] | - the meta-analysis included six studies, which yielded 10 independent effect sizes and a total of 1829 female participants ranging from 10 to 46 years. | - social media usage was positively correlated with internalization with a thin ideal | |
| Flynn et al., 2020 [182] | - 253 women (mean age = 37.05) completed the Eating Disorder Inventory Body Dissatisfaction Subscale, questions on participants' perception of thinness discrepancies, age, and BMI | - after controlling for age and BMI, perceived discrepancy between one's thinness level vs. the thinness women on social media find ideal is associated with women’s body dissatisfaction more than discrepancies with any other comparison group (i.e. close female friends, close male friends, men on social media) | |
| Lonergan et al., 2020 [213] | - 4209 adolescents (53.15% girls) completed scales on self-report photo investment (i.e. invested effort in posting a selfie to post on social media and monitoring its feedback), manipulation (i.e. extend to which participants edit their selfies prior to posting online) scales, avoidance of posting selfies to social media, investment in others’ selfies (i.e. extent to which the individual examine and interact with other peoples’ selfies), Eating Disorder Examination Questionnaire, extreme weight control behaviors, weight loss in the past 4 weeks, the K10 Psychological  Distress Scale, and the Pediatric Quality of  Life Scale Short Form 15 | Avoidance of posting selfies and photo manipulation were associated with greater  adjusted odds of meeting criteria for clinical/subclinical anorexia  Investment in others' selfies was associated with greater adjusted odds of meeting criteria for all eating disorder groups (bulimia, binge-eating disorder, night eating syndrome, unspecified feeding and eating disorder) except clinical and subclinical anorexia nervosa  and purging disorder.  Adolescent boys were more  likely to meet criteria for clinical and subclinical anorexia nervosa in the context of increasing avoidance of posting selfies | |
